# Measuring Quality of Maternal and Newborn Care in Developing Countries Using Demographic and Health Surveys

**DOI:** 10.1371/journal.pone.0157110

**Published:** 2016-06-30

**Authors:** Zoe Dettrick, Hebe N. Gouda, Andrew Hodge, Eliana Jimenez-Soto

**Affiliations:** The University of Queensland, School of Public Health, Public Health Building, Herston Road, Herston, Brisbane, Queensland, 4006, Australia; University of São Paulo, BRAZIL

## Abstract

**Background:**

One of the greatest obstacles facing efforts to address quality of care in low and middle income countries is the absence of relevant and reliable data. This article proposes a methodology for creating a single “Quality Index” (QI) representing quality of maternal and neonatal health care based upon data collected as part of the Demographic and Health Survey (DHS) program.

**Methods:**

Using the 2012 Indonesian Demographic and Health Survey dataset, indicators of quality of care were identified based on the recommended guidelines outlined in the WHO Integrated Management of Pregnancy and Childbirth. Two sets of indicators were created; one set only including indicators available in the standard DHS questionnaire and the other including all indicators identified in the Indonesian dataset. For each indicator set composite indices were created using Principal Components Analysis and a modified form of Equal Weighting. These indices were tested for internal coherence and robustness, as well as their comparability with each other. Finally a single QI was chosen to explore the variation in index scores across a number of known equity markers in Indonesia including wealth, urban rural status and geographical region.

**Results:**

The process of creating quality indexes from standard DHS data was proven to be feasible, and initial results from Indonesia indicate particular disparities in the quality of care received by the poor as well as those living in outlying regions.

**Conclusions:**

The QI represents an important step forward in efforts to understand, measure and improve quality of MNCH care in developing countries.

## Background

Poor quality of care is a major impediment to efforts aimed at improving the health of populations in developing countries, particularly with respect to Maternal, Neonatal and Child Health (MNCH) [1, 2]. Most recently, poor quality of care has been implicated in the disappointing outcomes of large scale programs aimed at increasing the coverage of maternal health services in developing countries including, the Janani Suraksha Yojana (JSY) conditional cash transfer program in India [3] and the Jamkesmas social insurance program in Indonesia [4]. It is suspected that while coverage of health services for these populations has increased, the quality of care provided is substandard[5]. Development of suitable measurement tools is necessary to support the improvements in quality of care and improve population health outcomes.

One of the greatest obstacles facing such efforts however is the current lack of data relating to quality, especially in low and middle income countries (LMICs) [6]. In the absence of fully functional health information systems, evidence on quality of care is scarce, and is often only available when specialised studies are conducted [7]. Additionally, there are multiple definitions of health care quality encompassing a multitude of dimensions ranging from efficacy and patient safety through to system efficiency and cultural appropriateness of care [8].There is as yet no international standard in how to measure quality of care, and as a result, differing definitions and choice in indicators limit comparability between studies [7].

The existing measures of quality of maternal care are typically focused on high level facility based care with caesarean and episiotomy rates [9], maternal near miss events [10], and maternal mortality commonly reported. Even fewer measures of quality of care exist for neonates, and the few that are commonly reported also emphasise tertiary level care. Consequently, existing measures tend to exclude women who deliver at home or in smaller clinics; in many developing contexts this can represent the majority of the population. The availability of data may also be hindered by the existence of largely unregulated private sectors that provide a high proportion of maternal and neonatal health services [11–14].

Given this situation, there has been interest in the use of surveys to collect information on people’s experiences of MNCH services. The collection of detailed population level data relating to multiple dimension of quality of care, such as provider actions and patient satisfaction, has been conducted using specially constructed surveys over small populations [15–18], but at the same time the availability of quality related measures in large scale population surveys has been limited. Attempts to use such surveys to report population level indicators of quality have been almost solely based on the basic coverage of antenatal care as reported by country level Demographic and Health Surveys (DHS) [19–21]. There is potential to increase the number of quality related indicators included in such surveys [22, 23], and more recent surveys do collect additional data related to care during and after delivery, but as of yet few studies have utilised these indicators within the context of examining quality of care.

This article proposes a methodology for creating a single “Quality Index” (QI) representing quality of maternal and neonatal health care based upon data collected as part of the DHS program and standard econometric techniques used to create composite indexes in other areas of development studies. As a result of the standardised, modular nature of all surveys conducted through the DHS program, existing health indicators generated by DHS surveys can be reliably compared across countries, and, in countries that regularly conduct these surveys every three to five years, over time. DHS surveys thus have great potential in relation to the estimation and monitoring of quality of care should it be feasible to derive such estimates from the available data. The 2012 Indonesia DHS [24] will be used as an example to demonstrate the viability of the method, as well as the potential for analysis of differences in quality of care.

## Methods

### Ethics approval and consent to participate

This research used pre-anonymized quantitative datasets, for which ethical approval was given at the time of data collection. The data was collected with the intention of being used for future research, and only quantitative responses to pre-approved questions are recorded in the dataset.

### Data sources

With the introduction of the Phase 6 DHS questionnaire in 2008, several additional variables related to the timing and content of particular actions during pregnancy and in the immediate postnatal period were included in the survey design. These questions were asked with regards to the last pregnancy experienced by all women with a live birth in the past five years, providing an opportunity to measure indicators associated with the quality of routine pregnancy and delivery care. The Indonesia 2012 dataset was chosen as it represents a large Phase 6 survey that included a number of questions relating to the content and timing pregnancy and delivery care. Comparison of measures derived from the standard DHS questions and those derived from all available questions would thus provide a test of the adequacy of the existing DHS questions in creating indicators of quality care.

### Selection of indicators

The Donabedian conceptual framework considers “quality” as the combination of structural elements (affecting the context in which care is delivered), process elements (all the actions that make up health care) and outcome elements (the effects of healthcare on the population) [25]. However, as standard DHS do not contain questions related to patient satisfaction, or to health inputs or outcomes, the definition of quality to be used for this analysis by necessity must be based on process indicators representing actions taken during contact with the health services in question. As a result, indicators were identified based on the recommended actions outlined in the WHO’s Integrated Management of Pregnancy and Childbirth (IMPAC) guidelines [26]. These guidelines are designed to outline essential practices by front line workers that address key areas of maternal and perinatal health programs. As such, they provide an objective, albeit heavily service oriented, framework on which to base indicator selection.

Based on these guidelines both the standard DHS questionnaire and the Indonesia 2012 questionnaire were examined for the presence of questions that could be used to construct relevant indicators of quality care. Table 1 shows the final set of indicators chosen for this analysis: a full list of all the potential indicators identified, including those not available in the Indonesia 2012 dataset are provided in S1 File with a rationale for each indicator’s use.

**Table 1:** Description of variables included in index construction.

**Indicator**	**Brief Rationale**
ANC visit in 1st Trimester	**A minimum of 4 ANC visits are recommended for all women; one in each of the 1**^**st**^ **and 2**^**nd**^ **trimesters, and two in the 3**^**rd**^ **trimester**
**ANC visit in 2nd Trimester**	
**ANC visits in 3rd Trimester**	
***Weight measured during ANC***	*In order to detect and appropriately treat issues that may affect maternal health*, *it is recommended that several diagnostic tests be undertaken as parts of ANC*. *Additionally Indonesian government guidelines specify that women should receive a “MNCH book” to keep track of health visits*
***Height measured during ANC***	
***Blood Pressure measured during ANC***	
***Urine sample taken during ANC***	
***Blood sample taken during ANC***	
***Stomach examined during ANC***	
***Consultation during ANC***	
***Received MNCH book during ANC***	
**Iron Supplementation during pregnancy**	**Appropriate preventative care may reduce both mortality and morbidity due to anaemia and tetanus infection**
**Tetanus Immunisation**	
***Pregnancy complication Advice***	*In order to prevent delays in care*, *women should be counselled about potential symptoms of pregnancy complications and the need for an appropriate birth plan*.
***Discussed place of delivery during pregnancy***	
***Discussed transportation to place of delivery during pregnancy***	
***Discussed who would assist delivery during pregnancy***	
***Discussed payment for delivery during pregnancy***	
***Discussed possible blood donor during pregnancy***	
**Baby was weighed at birth**	**Both maternal and neonatal health should be checked immediately following birth, and regularly thereafter. These checks should be used identify and treat potential complications as well as providing appropriate health advice and preventative care.**
**Baby was breastfed within 1 hr of birth**	
**No liquids given before milk began to flow (no prelacteal feed)**	
**Maternal postnatal check**	
**Neonatal postnatal check**	
**Postpartum Vitamin A within 2 months of delivery**	

### Data preparation

The sample was first limited to women of reproductive age with at least one live birth in the past five years. Due to difficulties in reconciling different populations at risk, childhood healthcare was omitted from the analysis, and the unit of observation was the mother and her lastborn child (the postnatal experience of the child was considered as a continuation of the mother’s experience during pregnancy and birth). Where possible, indicators were transformed into binary variables taking a value of either 0 (not present) or 1 (present). Observations with missing data for any of the indicators were excluded from the analysis, however in order to minimise the impact of missing observations, particularly from under-sampled areas, the following assumptions were made prior to data being dropped: Firstly, for variables related to yes/no questions, a response of “don’t know” was treated the same as a “no” response. This assumption does potentially increase the risk of recall bias affecting the sample, and creates a more conservative estimate, however unless there is a large proportion of cases where this response is prevalent it is unlikely to have a major effect on the overall validity of the sample. Secondly, for indicators where a quantitative value such as timing or quantity of service provided is missing or coded as “don’t know”, but other variables indicate that the service did occur, the observation was given the sample mean value of the quantitative variable. This approach is less likely to exclude observations for which recall bias hinders accurate quantification and is unlikely to be problematic unless a large proportion of observations are missing in this data.

### Index construction

One of the most important considerations in the construction of any composite index is the use of indicator weights to determine the final score.

The simplest option is to apply equal weighting, where all indicators contribute equally to the index and the final score is a simple average of all indicators. For example, the “Skilled Attendance Index” proposed by Hussein and colleagues [27] consisted of a score representing the simple percentage of 43 predetermined criteria met by that delivery (based on facility records). However this example also demonstrates one of the major limitations of equal weighting, as using equal weighting the provision of routine oxytocics contributed the same amount to the index as recording whether or not the patient started labour.

Another method of deriving weights is through the use of a statistical analysis of the dataset itself. The most commonly used technique is Principal Components Analysis (PCA)—a multivariate statistical technique that uses the correlation between multiple variables to determine the presence of coherent subsets of variables that may collectively represent an underlying factor (such as household wealth or social development) that cannot be directly measured [28]. These underlying factors, or “components”, are ordered such that the first component explains the largest possible amount of variation in the sample, the second (uncorrelated) components explaining additional variation, and with further components explaining progressively less and less variation. Examples of indexes using PCA derived weights include the Wealth Index [29] and the Indices of Social Development [30].

The most direct method of creating weights from the results of PCA is to assume that the first component corresponds to the underlying process that the index is attempting to measure [31, 32].

An index is then created by calculating a score for each observation consisting of the sum of the variable values multiplied by the calculated weight. The index produced by this method will be a relative one–as the index is based on the unique properties of the dataset itself, the resulting scores are not comparable between datasets. Likewise, it is possible that the principal components may vary between subgroups within the dataset–rural populations may have a different asset profile to those in urban areas. PCA derived indexes may therefore be of limited use in producing cross country comparisons, but are well suited for examining within country differences. In contrast the use of equal or theoretically derived weights provides a clearly understood measure that can be compared over different datasets; however the index will not be sensitive to changes in the relative importance of different variables in different contexts. For this reason two methods of indicator weighting were chosen for use in this analysis–one based on PCA derived weights, and a second based on a modified version of equal weighting.

The Equal Weight (EW) indices use a slight modification to equal weighting, similar to the theoretical component method used by the Human Development Index [30]. All original indicators carried equal weight in the final index; however indicators that did not take a binary form (that is, indicators where multiple levels of quality may exist) were treated as if made up of equally weighted subcomponents. This allowed for some level of discrimination between different levels of coverage for given indicators, while keeping to the equal weighting principle.

### Testing of indices

Four QI were constructed as a starting point of the analysis. These indices were based on the complete and DHS standard set of indicators and employed both the EW and PCA weighting methodologies. Additional indices were then created to determine if the number of categories for non-binary indicators, or the presence of absence of particular indicators, affected the robustness of the results. The indices were tested for internal coherence and robustness, as well as their comparability with each other, using the example set by Pritcher and Filmer [31]with regards to the development of the wealth index. In addition, Cronbach’s alpha was calculated as a measure of internal consistency for each set of indicators, with a coefficient of 0.7 or above considered to be acceptable[33]. Finally, a single QI was chosen to explore the variation in index scores across a number of known equity markers in Indonesia, including wealth, urban rural status and geographical region.

## Results

The 2012 Indonesia DHS originally provided a sample size of 15,262 women who had had at least one live birth in the five years prior to the survey. Following the initial construction of the indicator variables and the application of the stated imputation processes to deal with missing values, a total of 14,864 observations were included in the final analysis. Table 2 provides a breakdown of observations with no missing data, observations with at least one imputed variable and observations that were dropped due to missing data, by selected demographic factors. Two proportion z-tests were used to compare the imputed and missing observations to those with no missing data; there are no significant differences between the non-missing and dropped observations with the exception of wealth, with the dropped observations containing a higher proportion of observations from the poorest wealth quintile. In contrast, the imputed observations do appear to vary substantially from the non-missing observations with regards to urban rural residence, education and wealth. As these observations account for nearly 13% of the total sample, their omission from the remainder of the analysis might affect the representative ability of the dataset as a whole. Following a sensitivity analysis (S2 File) a decision was made to include these observations in the remainder of the analysis.

**Table 2:** Distribution of observations with imputed or missing data.

Indicator	*Non Missing*	*Imputed*	*Dropped*	Indicator	*Non Missing*	*Imputed*	*Dropped*
***Age***				***Wealth Quintile***			
**15–19**	472	54	21	**Poorest**	3,721	499	151
***%***	*3*.*65*	*2*.*82*	*5*.*28*	***%***	*28*.*74*	*26*.*03*	*37*.*94*
**20–24**	2,520	346	71	**Poorer**	2,681	343	77
***%***	*19*.*46*	*18*.*05*	*17*.*84*	***%***	*20*.*71*	*17*.*89*	*19*.*35*
**25–29**	3,592	520	102	**Middle**	2,387	371	58
***%***	*27*.*74*	*27*.*13*	*25*.*63*	***%***	*18*.*44*	*19*.*35*	*14*.*57*
**30–34**	3,116	474	87	**Richer**	2,227	348	69
***%***	*24*.*07*	*24*.*73*	*21*.*86*	***%***	*17*.*2*	*18*.*15*	*17*.*34*
**35–39**	2,147	329	65	**Richest**	1,931	356	43
***%***	*16*.*58*	*17*.*17*	*16*.*33*	***%***	*14*.*91*	*18*.*57*	*10*.*8*
**40–44**	916	162	42	***p-value***		*0*	*0*.*001*
***%***	*7*.*07*	*8*.*45*	*10*.*55*	**Island Group**			
**45–49**	184	32	10	**Sumatera**	3,730	661	93
***%***	*1*.*42*	*1*.*67*	*2*.*51*	***%***	*28*.*81*	*34*.*48*	*23*.*37*
***p-value***		*0*.*091*	*0*.*025*	**Java**	3,151	441	71
***Residence***				***%***	*34*.*34*	*23*	*17*.*84*
**Urban**	5,887	941	166	**Bali and Nusa Tenggara**	1,257	63	19
***%***	*45*.*47*	*49*.*09*	*41*.*71*	***%***	*9*.*71*	*3*.*29*	*4*.*77*
**Rural**	7,060	976	232	**Kalimantan**	1,433	183	37
***%***	*54*.*53*	*50*.*91*	*58*.*29*	***%***	*11*.*07*	*9*.*55*	*9*.*3*
***p-value***		*0*.*003*	*0*.*138*	**Sulawesi**	2,111	309	124
***Education***				***%***	*16*.*3*	*16*.*12*	*31*.*16*
**No education**	324	85	17	**Maluku and Papua**	1,265	260	54
***%***	*2*.*5*	*4*.*43*	*4*.*27*	***%***	*9*.*77*	*13*.*56*	*13*.*57*
**Primary**	4,052	529	128	***p-value***		0	0
***%***	*31*.*3*	*27*.*6*	*32*.*16*				
**Secondary**	6,909	1,033	213				
***%***	*53*.*36*	*53*.*89*	*53*.*52*				
**Higher**	1,662	270	40				
***%***	*12*.*84*	*14*.*08*	*10*.*05*				
***p-value***		*0*	*0*.*067*				
***Total***	**12,947**	**1,917**	**398**	*** ***	** **	** **	** **
**% of sample**	*84*.*8%*	*12*.*6%*	*2*.*6%*	** **	* *	* *	* *

Table 3 provides a full list of the initial categorisation used to create variables for index construction. The table also reports the mean and standard deviation of each variable. Coverage of different indicators varied substantially; some, such as stomach examination and blood pressure measurement, were over 90%, while other such as discussion about blood donors were quite low. In general however coverage of the indicators was high enough to allow for meaningful differentiation between high and low levels of quality.

**Table 3:** Summary of Initial Indicator Categorisation and Means.

Indicator	Categories	Indicator Set	Mean	[95% Conf. Interval]	
ANC visit in 1st Trimester		DHS	0.7586	*0*.*7517*	*0*.*7655*
ANC visit in 2nd Trimester		Indonesia	0.8990	*0*.*8942*	*0*.*9039*
ANC visits in 3rd Trimester	1	Indonesia	0.0094	*0*.*0079*	*0*.*0110*
	2		0.8459	*0*.*8401*	*0*.*8517*
	None		0.1447	*0*.*1391*	*0*.*1504*
Weight measured during ANC		Indonesia	0.8932	*0*.*8883*	*0*.*8982*
Height measured during ANC		Indonesia	0.4395	*0*.*4315*	*0*.*4475*
Blood Pressure measured during ANC		DHS	0.9087	*0*.*9041*	*0*.*9133*
Urine sample taken during ANC		DHS	0.4131	*0*.*4052*	*0*.*4210*
Blood sample taken during ANC		DHS	0.3919	*0*.*3840*	*0*.*3997*
Stomach examined during ANC		Indonesia	0.9378	*0*.*9340*	*0*.*9417*
Consultation during ANC		Indonesia	0.7910	*0*.*7844*	*0*.*7975*
Received MNCH book during ANC		Indonesia	0.7746	*0*.*7679*	*0*.*7813*
Iron Supplementation during pregnancy	1–29 days	DHS	0.2042	*0*.*1977*	*0*.*2107*
	30–89 days		0.2548	*0*.*2478*	*0*.*2619*
	90–179 days		0.1204	*0*.*1151*	*0*.*1256*
	180–269 days		0.1015	*0*.*0967*	*0*.*1064*
	270+ days		0.0480	*0*.*0445*	*0*.*0514*
	None		0.2711	*0*.*2640*	*0*.*2783*
Tetanus Immunisation	Full Protection	DHS	0.6154	*0*.*6076*	*0*.*6232*
	Partial Protection		0.1699	*0*.*1638*	*0*.*1759*
	None		0.2147	*0*.*2081*	*0*.*2213*
Pregnancy complication Advice	Symptoms only	DHS	0.0237	*0*.*0212*	*0*.*0261*
	Symptoms and Help		0.4649	*0*.*4569*	*0*.*4729*
	None		0.5114	*0*.*5034*	*0*.*5195*
Discussed place of delivery during pregnancy		Indonesia	0.7879	*0*.*7814*	*0*.*7945*
Discussed transportation to place of delivery during pregnancy		Indonesia	0.5779	*0*.*5700*	*0*.*5858*
Discussed who would assist delivery during pregnancy		Indonesia	0.7863	*0*.*7797*	*0*.*7929*
Discussed payment for delivery during pregnancy		Indonesia	0.7432	*0*.*7362*	*0*.*7502*
Discussed possible blood donor during pregnancy		Indonesia	0.1590	*0*.*1532*	*0*.*1649*
Baby was weighed at birth		DHS	0.8702	*0*.*8648*	*0*.*8756*
Baby was breastfed within 1 hr of birth		DHS	0.4826	*0*.*4746*	*0*.*4907*
No liquids given before milk began to flow (no prelacteal feed)		DHS	0.3786	*0*.*3708*	*0*.*3864*
Maternal postnatal check	<2hrs	DHS	0.4732	*0*.*4651*	*0*.*4812*
	3–12 hrs		0.1792	*0*.*1730*	*0*.*1853*
	13-24hrs		0.1142	*0*.*1091*	*0*.*1194*
	25-48hrs		0.0225	*0*.*0201*	*0*.*0249*
	49hrs +		0.0780	*0*.*0737*	*0*.*0823*
	None		0.1330	*0*.*1275*	*0*.*1385*
Neonatal postnatal check	<2hrs		0.2926	*0*.*2853*	*0*.*2999*
	3–12 hrs		0.0953	*0*.*0905*	*0*.*1000*
	13-24hrs		0.0747	*0*.*0705*	*0*.*0790*
	25-48hrs		0.0184	*0*.*0163*	*0*.*0206*
	49hrs +		0.1653	*0*.*1593*	*0*.*1713*
	None		0.3537	*0*.*3460*	*0*.*3614*
Postpartum Vitamin A within 2 months of delivery		DHS	0.4624	*0*.*4544*	*0*.*4704*

Table 4 reports the PCA derived variable weights for a number of scenarios in which the included indicators, and the number of quality levels for each indicator, differ. In the initial scenario (column 1), all potential indicators were included and up to five categories of quality were available for each indicator. The results are as expected–variables such as “No ANC visits in third trimester” and “No Tetanus Protection” are strongly negative while their counterparts thought to represent a high level of quality (“2+ ANC visits in third trimester” and “Full Tetanus Protection”) score quite positively. Interestingly, the variables with the strongest effect on the final quality score are those related to interpersonal communication during pregnancy; advice on pregnancy complications scored particularly highly, as did discussion of transportation, place and payment for delivery. The results of the same PCA process carried out only on core DHS indicators is shown in column 2. The driving variables remain roughly the same, however the reduction in the number of variables has increased the magnitude of the weights assigned to the remaining indicators. Pregnancy complication advice remains the highest scored variable, but the influence of urine and blood tests during ANC and provision of timely postnatal care becomes more apparent.

**Table 4:** Variable weights for PCA indexes using different indicator sets and categorisation.

Indicators			Scenario 1	2	3	4	5	6	7	8
			All Indicators, <5 categories +prelacteal feeding	DHS Indicators, <5 categories +prelacteal feeding	All Indicators, <5 categories	DHS Indicators, <5 categories	All Indicators, <3 categories	DHS Indicators, <3 categories	All indicators, < 3 categories Any iron	DHS indicators, < 3 categories Any iron
ANC visit in 1st Trimester			0.167	0.193	0.167	0.193	0.164	0.185	0.168	0.192
ANC visit in 2nd Trimester			0.152		0.152		0.150		0.152	
ANC visits in 3rd Trimester		1	-0.012		-0.012		-0.012		-0.012	
		2	0.188		0.188		0.186		0.189	
		None	-0.176		-0.176		-0.174		-0.177	
Weight measured during ANC			0.172		0.172		0.171		0.173	
Height measured during ANC			0.190		0.191		0.186		0.192	
Blood Pressure measured during ANC			0.156	0.162	0.156	0.162	0.155	0.161	0.156	0.160
Urine sample taken during ANC			0.202	0.263	0.202	0.263	0.196	0.248	0.203	0.266
Blood sample taken during ANC			0.171	0.220	0.171	0.221	0.166	0.207	0.172	0.223
Stomach examined during ANC			0.122		0.122		0.122		0.123	
Consultation during ANC			0.194		0.194		0.190		0.195	
Received MNCH book during ANC			0.199		0.199		0.197		0.200	
Iron Supplementation during pregnancy										
	Full (270+ days)		0.025	0.031	0.025	0.031	0.020	0.019	0.197	0.244
	Partial	1–29 days	0.009	0.012	0.009	0.012	0.200	0.279		
		30–89 days	0.065	0.088	0.064	0.088				
		90–179 days	0.049	0.061	0.049	0.061				
		180–269 days	0.054	0.066	0.054	0.066				
	None		-0.201	-0.257	-0.201	-0.257	-0.220	-0.298		
Tetanus Immunisation										
		Full Protection	0.206	0.285	0.207	0.285	0.206	0.279	0.207	0.285
		Partial Protection	-0.020	-0.047	-0.020	-0.047	-0.020	-0.044	-0.020	-0.047
		None	-0.186	-0.238	-0.186	-0.238	-0.186	-0.235	-0.187	-0.238
Pregnancy complication Advice										
		Symptoms only	0.000	0.000	0.000	0.000	0.000	0.000	0.000	0.000
		Symptoms and Help	0.270	0.396	0.270	0.396	0.263	0.369	0.272	0.401
		None	-0.270	-0.396	-0.270	-0.396	-0.263	-0.369	-0.272	-0.401
Discussed place of delivery			0.216		0.216		0.210		0.218	
Discussed transportation			0.255		0.255		0.246		0.257	
Discussed who would assist delivery			0.204		0.204		0.198		0.206	
Discussed payment for delivery			0.214		0.213		0.207		0.215	
Discussed possible blood donor			0.107		0.107		0.103		0.108	
Baby was weighed at birth			0.156	0.177	0.156	0.177	0.153	0.170	0.156	0.176
Baby was breastfed within 1 hr of birth			0.029	0.049	0.032	0.051	0.030	0.047	0.031	0.053
No liquids given before milk began to flow			-0.023	-0.010						
Maternal postnatal check										
	Full (<2hrs)		0.147	0.218	0.147	0.218	0.140	0.205	0.146	0.233
	Partial	3–12 hrs	0.025	0.011	0.024	0.011	0.009	-0.028	0.005	-0.051
		13-24hrs	-0.005	-0.018	-0.005	-0.018				
		25-48hrs	-0.004	-0.007	-0.004	-0.007				
		49hrs +	-0.012	-0.020	-0.012	-0.020				
	None		-0.151	-0.184	-0.151	-0.184	-0.148	-0.177	-0.151	-0.182
Neonatal postnatal check										
	Full (<2hrs)		0.140	0.215	0.140	0.215	0.133	0.199	0.137	0.219
	Partial	3–12 hrs	0.018	0.017	0.018	0.017	0.045	0.054	0.044	0.042
		13-24hrs	0.007	0.005	0.007	0.005				
		25-48hrs	0.001	0.002	0.001	0.002				
		49hrs +	0.011	0.017	0.012	0.017				
	None		-0.178	-0.256	-0.178	-0.256	-0.179	-0.252	-0.181	-0.261
Postpartum Vitamin A			0.155	0.233	0.155	0.233	0.154	0.225	0.156	0.235
Rho			0.1752	0.1589	0.1814	0.1669	0.2017	0.1969	0.2035	0.1978
*Cronbach’s α*			*0*.*8359*	*0*.*6877*	*0*.*8411*	*0*.*7020*	*0*.*8578*	*0*.*7369*	*0*.*8534*	*0*.*7248*

The variable representing no prelacteal feeding (believed to be an indicator of good quality care) has a negative score, reflecting the known decrease in exclusive breastfeeding in wealthier and more urbanised populations [34].Columns 3 and 4 report the results of the previous scenarios with the removal of prelacteal feeding as an indicator. There are no major changes in the weights assigned to other variables, and the overall proportion of variance explained by the first component increased only slightly, suggesting that the omission of this indicator did not overly affect the index.

Because it is possible that the number of categories used to define quality within a given indicator may affect overall representation of the indicator within the dataset, two additional scenarios were included, in which the levels of quality allowed for each indicator were limited to “Full”, “Partial” and “None” (columns 5 and 6). The change in classification only affected three indicators; iron supplementation during pregnancy, maternal PNC and neonatal PNC. For both PNC indicators the consolidation of the partial quality variables resulted in relatively little effect–however while having no iron supplementation carries a strongly negative weight as expected, “partial” iron supplementation carries a much higher positive weight than “full” iron supplementation. As the magnitude of the partial and no supplementation variables is considerable, complete exclusion of this variable was inappropriate and might reduce the explanatory ability of the index.

As such, a scenario in which a replacement indicator representing the presence of iron supplementation rather than its duration was created. The results of this scenario can be seen in last two columns in Table 4. Again, this change resulted in only minor increases in the variance explained by the principal component. This consistency in weights and variance explained by the principal component was also seen during sensitivity testing involving the recreation of a single QI (Scenario 5) using random subsamples of the dataset (see S2 File). With regards to internal consistency, only the DHS based index that included prelacteal feeding (in Column 2) had an unacceptably low alpha coefficient. This is understandable as Cronbach’s alpha is designed to reflect the degree to which the indicator set reflects a single, unidimensional construct and prelacteal feeding has already been identified as an outlier with regards to the other indicators. Overall these results suggest that the PCA based QIs were not overly sensitive to minor variation in the choice and classification of indicators; the greatest differences in results occurred as a result of the reduced number of variables in the standard DHS indicator set.

To test that this robustness extended also to indices created through the EW method, observations were ranked according to their scores measured using the PCA and EW indices for the scenarios mentioned above and divided into quintiles. The mean value for each variable was then compared according to each index; as an example, Table 5 shows the mean value for each variable according the PCA and EW index quintiles for the first scenario. A full table of these results is provided in S2 File. In general the results appear to be robust, with “positive” variables such as blood testing during ANC having higher means in the higher quintiles and “negative” variables such as no tetanus protection having higher means in the lower quintiles. Table 6 compares the correlation between quintiles created using QIs that utilise different indicator sets, categorisation and weighting methods. Despite the considerable variation in content the classification of observations into quintiles is relatively stable; even scenarios in which the underlying indicator set and weighting methodology differ have over 75% correlation. This suggests that the indices are reflecting differences in the underlying quality of care rather than differences in a subset of dominant indicators.

**Table 5:** Indicator Means by Quality Index based Quintiles (Scenario 1).

Variable		EW Quintile					PCA Quintile				
		Q1	Q2	Q3	Q4	Q5	Q1	Q2	Q3	Q4	Q5
ANC visit in 1st Trimester		0.436	0.741	0.817	0.865	0.942	0.443	0.734	0.809	0.864	0.944
ANC visit in 2nd Trimester		0.621	0.931	0.963	0.988	0.996	0.587	0.939	0.978	0.992	0.999
ANC visits in 3rd Trimester	1	0.037	0.006	0.002	0.001	0	0.044	0.003	0	0	0
	2	0.508	0.863	0.926	0.957	0.983	0.428	0.863	0.952	0.987	0.999
	None	0.455	0.131	0.072	0.042	0.017	0.528	0.134	0.048	0.013	0.001
Weight measured during ANC		0.555	0.936	0.985	0.995	1	0.538	0.945	0.987	0.997	1
Height measured during ANC		0.127	0.301	0.422	0.575	0.785	0.154	0.345	0.422	0.532	0.744
Blood Pressure measured during ANC		0.616	0.955	0.983	0.995	0.999	0.6	0.96	0.987	0.998	0.999
Urine sample taken during ANC		0.092	0.257	0.378	0.552	0.801	0.117	0.279	0.383	0.521	0.765
Blood sample taken during ANC		0.127	0.255	0.327	0.492	0.769	0.159	0.286	0.356	0.464	0.695
Stomach examined during ANC		0.726	0.98	0.994	0.993	0.999	0.718	0.979	0.994	0.998	1
Consultation during ANC		0.422	0.78	0.871	0.921	0.969	0.421	0.766	0.86	0.929	0.98
Received MNCH book during ANC		0.395	0.759	0.858	0.912	0.959	0.401	0.754	0.84	0.913	0.965
Iron Supplementation during pregnancy	Full (270+ days)	0.003	0.026	0.04	0.061	0.112	0.004	0.026	0.038	0.071	0.101
	Partial (1–29 days)	0.207	0.251	0.217	0.194	0.151	0.183	0.244	0.233	0.205	0.156
	(30–89 days)	0.151	0.277	0.291	0.284	0.273	0.129	0.264	0.284	0.308	0.289
	(90–179 days)	0.038	0.098	0.132	0.155	0.182	0.031	0.096	0.124	0.148	0.202
	(180–269 days)	0.016	0.053	0.104	0.145	0.192	0.013	0.047	0.096	0.139	0.212
	None	0.585	0.295	0.215	0.161	0.091	0.639	0.324	0.224	0.129	0.039
Tetanus Immunisation	Full Protection	0.298	0.558	0.662	0.736	0.832	0.267	0.537	0.645	0.743	0.884
	Partial Protection	0.175	0.21	0.181	0.165	0.116	0.176	0.22	0.199	0.161	0.094
	None	0.528	0.231	0.157	0.099	0.051	0.557	0.243	0.156	0.096	0.022
Pregnancy complication Advice	Symptoms only	0.019	0.037	0.027	0.021	0.014	0.017	0.036	0.028	0.025	0.012
	Symptoms and Help	0.098	0.318	0.474	0.643	0.806	0.096	0.269	0.429	0.628	0.903
	None	0.883	0.644	0.499	0.336	0.18	0.887	0.695	0.543	0.347	0.085
Discussed place of delivery		0.358	0.714	0.917	0.968	0.996	0.386	0.682	0.898	0.976	0.997
Discussed transportation		0.114	0.396	0.656	0.81	0.932	0.165	0.382	0.616	0.797	0.929
Discussed who would assist delivery		0.39	0.704	0.896	0.961	0.994	0.424	0.668	0.873	0.972	0.995
Discussed payment for delivery		0.333	0.656	0.845	0.919	0.976	0.364	0.626	0.821	0.927	0.978
Discussed possible blood donor		0.016	0.044	0.096	0.189	0.458	0.024	0.059	0.109	0.184	0.42
Baby was weighed at birth		0.56	0.884	0.946	0.975	0.992	0.547	0.871	0.954	0.981	0.998
Baby was breastfed within 1 hr of birth		0.36	0.38	0.436	0.531	0.712	0.446	0.457	0.461	0.499	0.549
No liquids given before milk began to flow		0.349	0.295	0.311	0.374	0.568	0.429	0.374	0.347	0.361	0.382
Maternal postnatal check	<2hrs	0.233	0.402	0.484	0.575	0.68	0.24	0.405	0.489	0.543	0.689
	3–12 hrs	0.111	0.186	0.209	0.212	0.18	0.103	0.179	0.21	0.223	0.181
	13-24hrs	0.116	0.139	0.127	0.11	0.079	0.102	0.135	0.134	0.121	0.079
	25-48hrs	0.03	0.032	0.023	0.015	0.012	0.027	0.035	0.02	0.022	0.009
	49hrs +	0.106	0.103	0.089	0.054	0.035	0.092	0.104	0.089	0.066	0.039
	None	0.403	0.138	0.068	0.035	0.015	0.436	0.144	0.057	0.025	0.003
Neonatal postnatal check	<2hrs	0.097	0.188	0.275	0.373	0.539	0.099	0.186	0.275	0.359	0.545
	3–12 hrs	0.051	0.092	0.107	0.114	0.114	0.049	0.09	0.1	0.128	0.109
	13-24hrs	0.059	0.084	0.082	0.083	0.066	0.052	0.08	0.086	0.086	0.069
	25-48hrs	0.017	0.02	0.023	0.018	0.015	0.013	0.023	0.021	0.02	0.016
	49hrs +	0.158	0.189	0.18	0.173	0.126	0.144	0.183	0.168	0.171	0.161
	None	0.618	0.427	0.333	0.24	0.141	0.643	0.439	0.35	0.236	0.1
Postpartum Vitamin A		0.211	0.348	0.446	0.581	0.736	0.238	0.378	0.447	0.55	0.698

**Table 6:** Correlation between quintiles using different Quality Indexes.

	Quality Index	A	B	C	D	E	F	G	H	I	J
A	All Indicators, <5 categories +prelacteal feeding, Equal Weighting	1									
B	DHS Indicators, <5 categories +prelacteal feeding, Equal Weighting	0.837	1								
C	All Indicators, <5 categories +prelacteal feeding, PCA Weighting	0.920	0.821	1							
D	DHS Indicators, <5 categories +prelacteal feeding, PCA Weighting	0.771	0.866	0.875	1						
E	All Indicators, <3 categories, Equal Weighting	0.888	0.795	0.864	0.754	1					
F	DHS Indicators, <3 categories, Equal Weighting	0.828	0.932	0.817	0.853	0.897	1				
G	All Indicators, <3 categories, PCA Weighting	0.863	0.787	0.902	0.832	0.974	0.885	1			
H	DHS Indicators, <3 categories, PCA Weighting	0.781	0.864	0.870	0.945	0.851	0.926	0.919	1		
I	All Indicators, <3 categories, Any Iron, PCA Weighting	0.933	0.823	0.972	0.855	0.871	0.818	0.898	0.853	1	
J	DHS Indicators, <3 categories, Any Iron, PCA Weighting	0.799	0.894	0.873	0.948	0.776	0.879	0.831	0.926	0.867	1

For the purpose of summarising differences in quality of care within Indonesia, a single QI was chosen. From a policy perspective, relative variation in quality of care is important in order to provide an overview of which groups are doing better or worse than their peers; for this reason the following sections utilise the PCA index based on all available indicators using simplified categorisation (Scenario 5 from Table 4).

**Fig 1:**
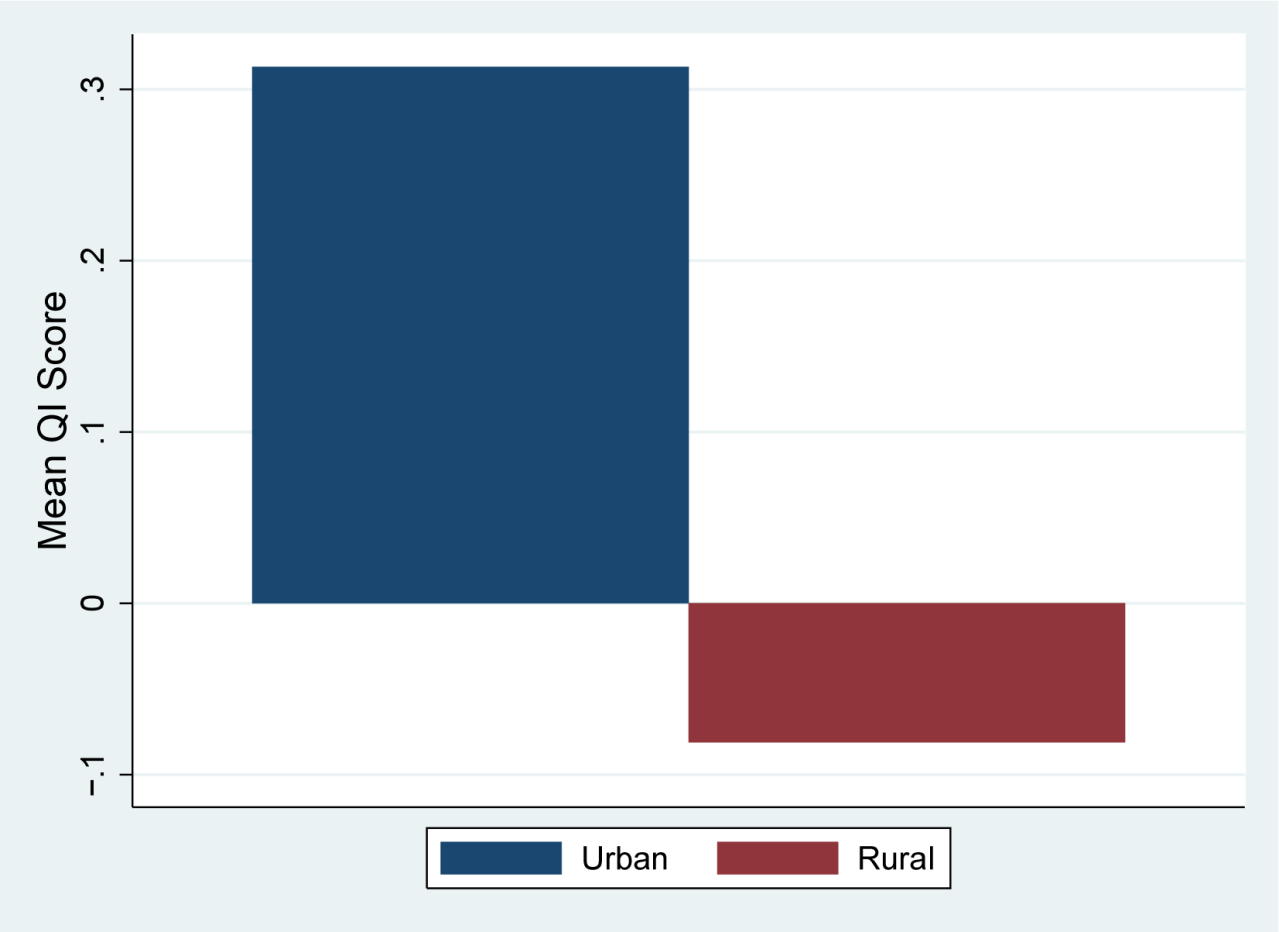
Quality Index score by Urban/Rural Status (National).

**Fig 2:**
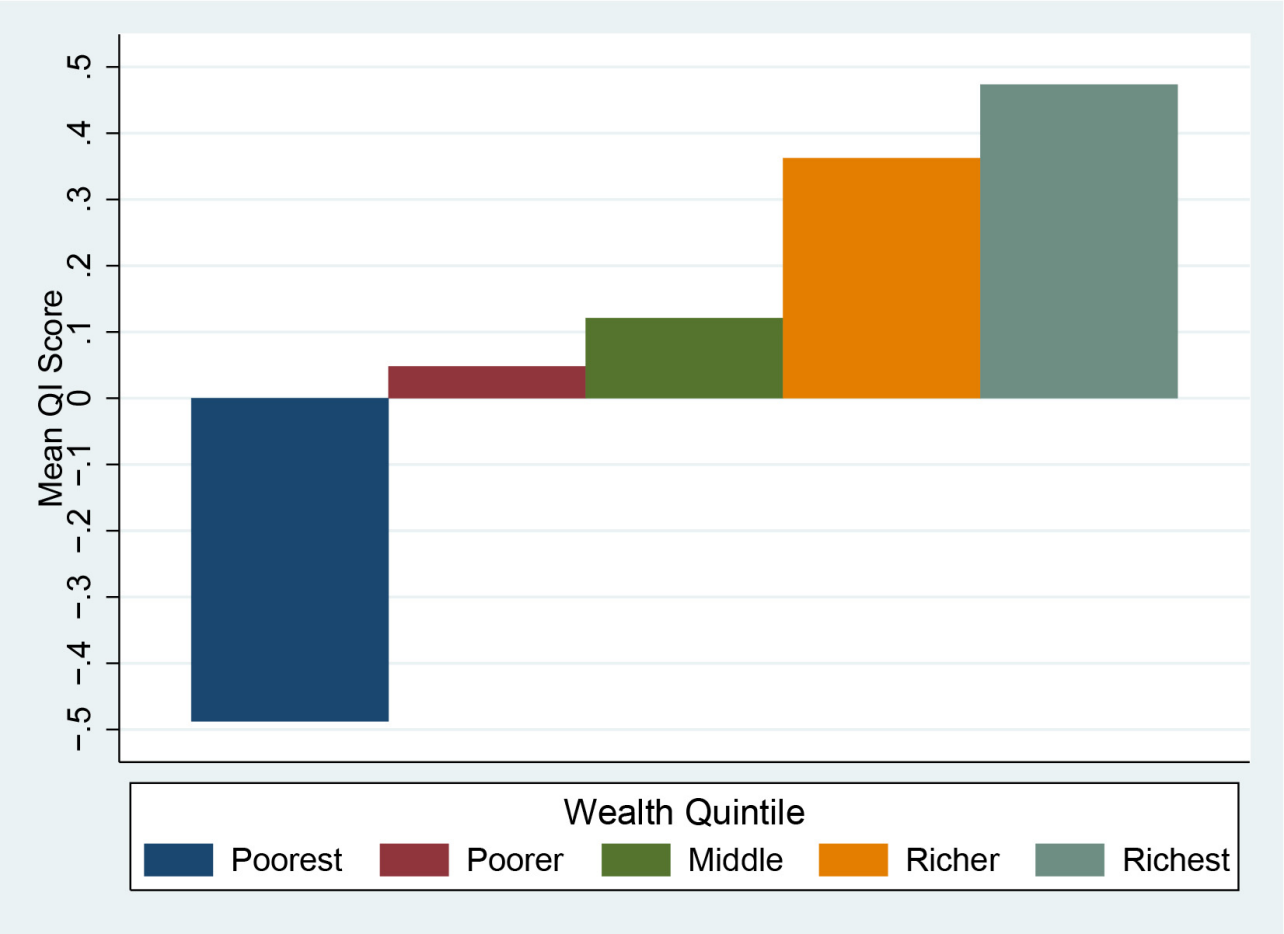
Quality Index score by Wealth Quintile (National).

Urban areas have a substantially higher score compared to rural areas (Fig 1). Given that rural areas are known to have issues with access to services[35], it is likely that these areas may have issues with key inputs that limit the level of quality available to the population. Wealth also appears to have an effect on quality–Fig 2 shows the mean scores by wealth quintile.

**Fig 3:**
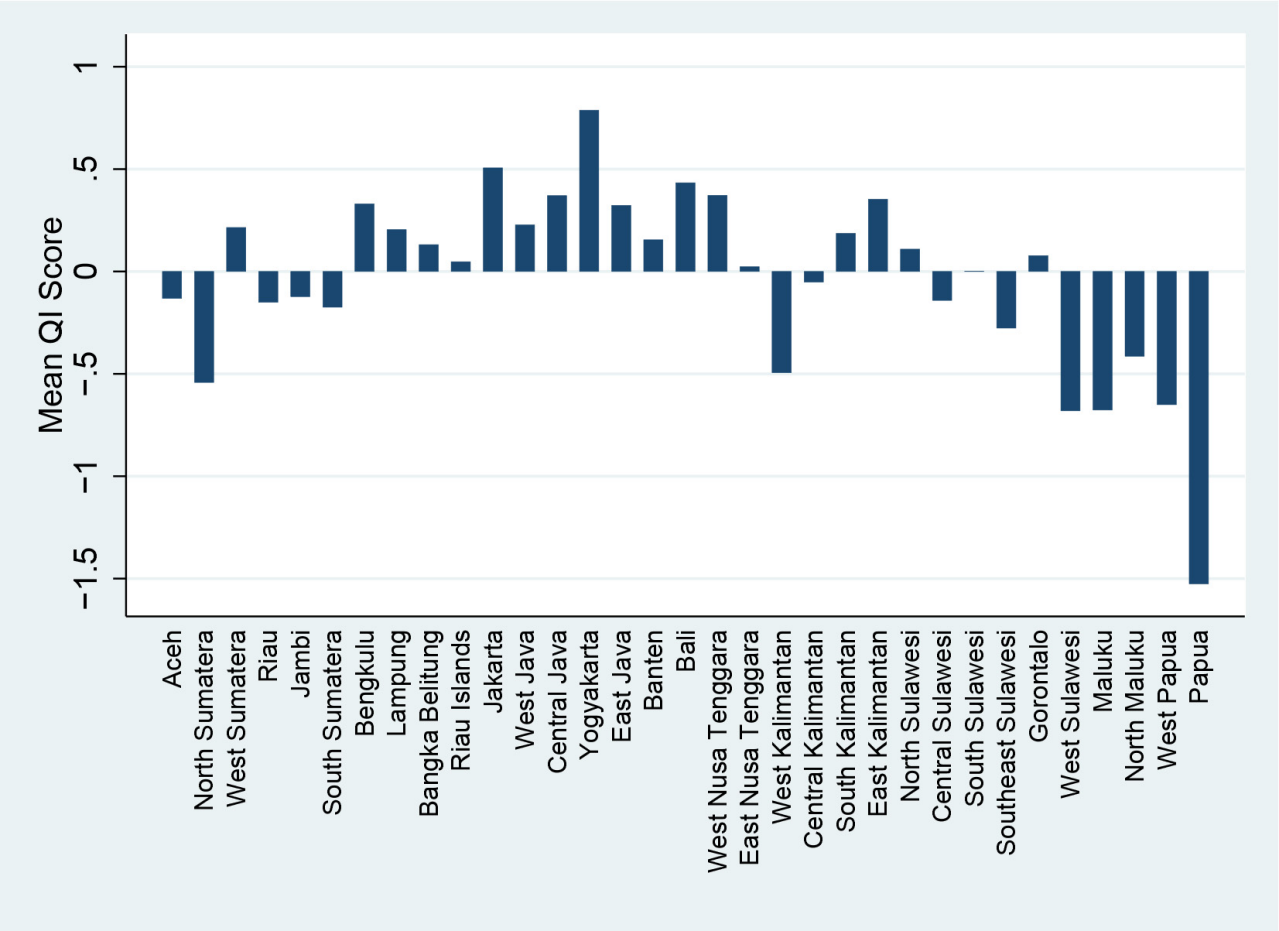
Quality Index Score by Province.

Indonesia is a large and diverse country, and health outcomes have been known to vary substantially between different geographic regions, and as seen in Fig 3 and quality of care is no exception. The impressively high score in Yogyakarta is overwhelmed by the massively negative score seen in Papua and provinces in the Java/Bali region have relatively good scores while outlying regions such as Sulawesi and Maluku are generally negative.

**Fig 4:**
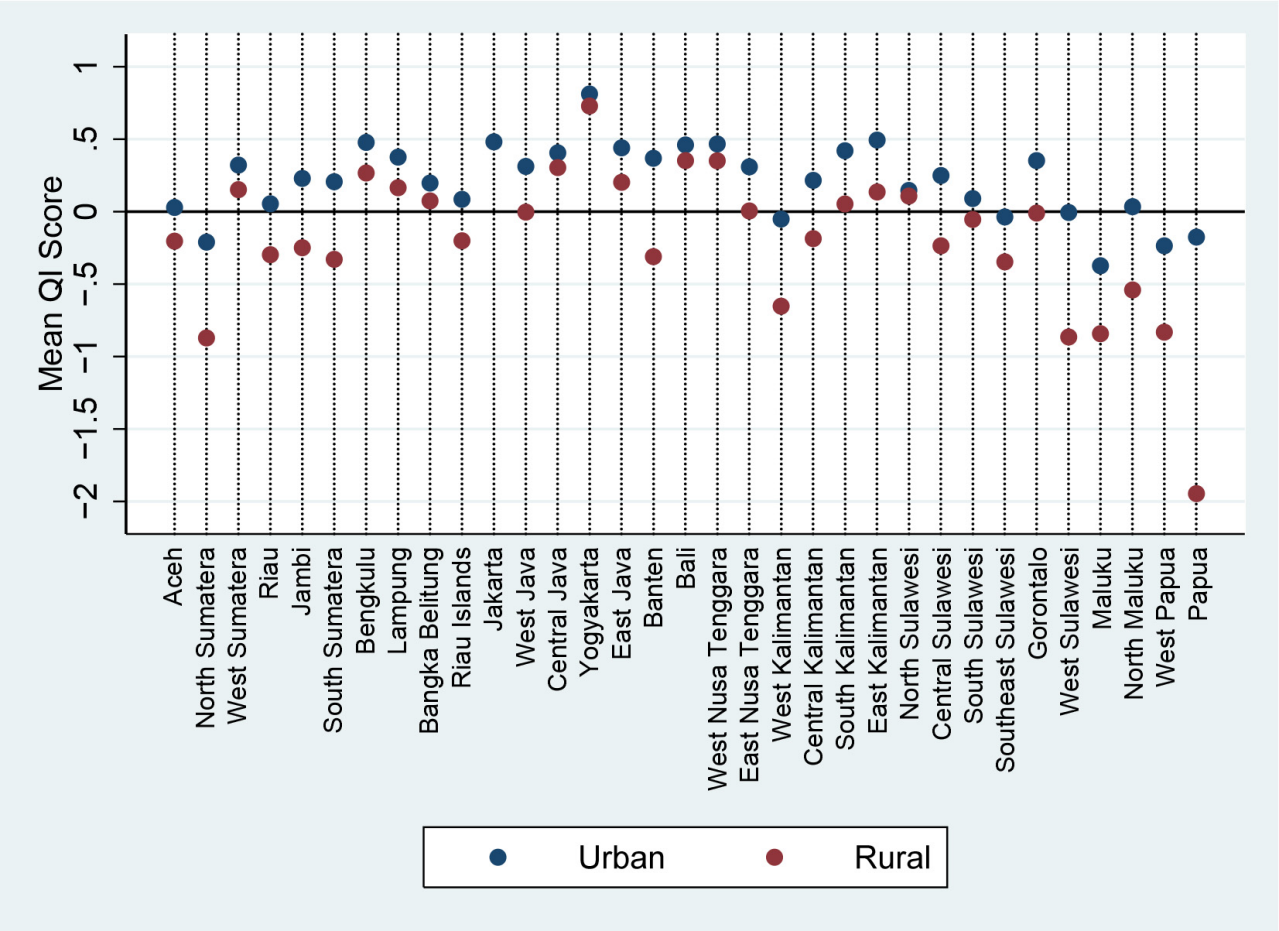
Quality Index Score by Province and Urban/Rural Status.

**Fig 5:**
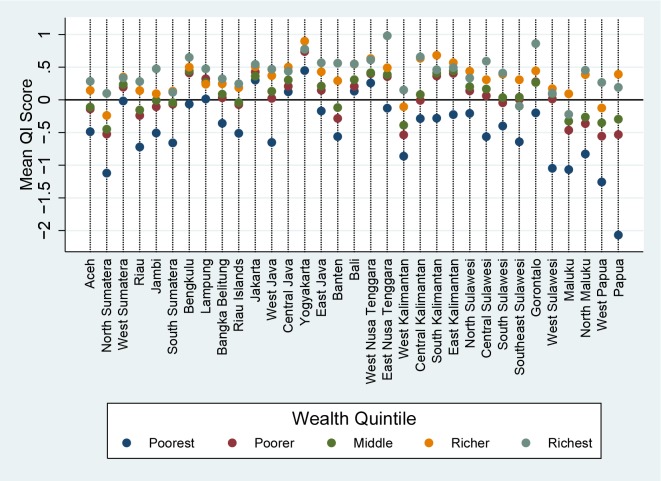
Quality Index Score by Province and Wealth Quintile.

Examining these provincial scores by urban rural status makes the situation even clearer. Fig 4 shows that in the provinces that are doing well there are only minor differences between rural and urban populations, while the rural population in these outlying regions appear to be receiving much worse care than their urban counterparts. As it is possible that this difference in urban/rural outcomes is due to differences in wealth between the two populations, Fig 5 illustrates the mean quality score for each wealth index by region. Perhaps unsurprisingly, the provinces showing great differences between urban and rural populations also show large differentials between the poorest and wealthiest quintiles. It is interesting however that even the wealthiest quintile in remote regions do not score highly; conversely in high performing regions such as Yogyakarta even the poorest appear to be receiving a high level of care.

## Discussion

This is the first study to utilise PCA based techniques to attempt to quantify variation in quality of care using DHS data. As one of the major assumptions of the PCA process is that the resulting index is capturing an underlying dimension of quality, it is heartening that both equal weighting and PCA weighting techniques produced indexes that were internally consistent and robust to the inclusion or exclusion of individual indicators. Similarly, the similarities between the results of the country-specific and DHS indicator sets suggests that the process may create similarly robust results in other countries where only the standard set of DHS questions were included.

There is concern however that the current methodology creates an index that is more reflective of access to care rather than the quality of care provided; a woman without access to health services might score the same as a woman with access to only very poor quality services. From a systemic perspective, lack of services may in fact an indicator of poor quality, but from a policy perspective it is far more useful to quantify quality within the scope of those who do receive care in some form. The difficulty lies in defining who does and does not have access to care: should availability of services be considered or only usage? Should the types of services available matter or is having access to any form of care enough? These are issues that should be addressed as the methodology is further refined.

Such refinement would be highly beneficial, as the use of a single composite index to allow the direct comparison of different population subgroups has allowed for greater understanding of the factors driving inequities in MNCH care. In particular, the differing patterns of quality scores seen between and within regions in Indonesia suggests that place of residence may have a greater influence on determining the quality of maternal and neonatal care than wealth or urban-rural status alone. This is perhaps unsurprising given the heavily decentralised nature of Indonesia’s health system; significant regional variation has been noted for other health related measures such as child mortality [36, 37], and observational data suggest that the performance of local health systems varies substantially from district to district. Countries with more highly centralised health systems would be expected demonstrate different patterns of variation. Importantly, as the questions used to construct the QI score are based around actions that should be undertaken as part of routine care by health providers, it is possible that these results may provide a mechanism through which poorly performing systems may be identified for policy intervention. For example, the positive association between having over a month of iron supplementation and other quality markers, but lack of association with having over six months of iron supplementation, suggests that the continuity of supplementation is an area in need of further attention.

This does however reveal another limitation of the current methodology; without indicators related to the types of health messages provided by health staff, it is difficult to determine why existing practices are ineffective and thus design an appropriate health system intervention to address the problem. In terms of health education, advice about recognising pregnancy complications during ANC is the sole indicator available as part of the standard DHS questionnaire, and while there was a very strong association between the QI and this indicator, as well as the Indonesia specific indicators relating to birth preparedness (discussion of which should occur as part of standard ANC) it remains unknown if other critical health messages communicated to the client. Given the acknowledged importance of interpersonal interaction with the health provider in encouraging continuance of care, through health promotion and education as well as patient satisfaction [38, 39], future QI would benefit from questions addressing these issues.

Another limitation of the current indicators is the lack of coverage for routine interventions such as clean delivery, thermal management active management of third stage of. Similarly, there is scant information relating to postnatal care–both in terms of content and timing of follow up visits. As the major benefits of PNC come from the prompt identification of issues that require additional care [40, 41] this represents an area that should ideally be included in any holistic measure of MNCH quality. Despite concerns regarding recall bias, questions relating to these interventions have occasionally been included in individual countries’ DHSs; for example, Nepal 2011 [42] included a questions about oxytocin use during delivery, Philippines 2013 [43] included questions about the type of examinations given during maternal PNC and Bangladesh 2011 [44] included questions about cord care and temperature management (albeit only for home deliveries). Similarly, questions about advice and counselling for specific MNCH issues have been included in a number of other country surveys[45]. While there is need for a consensus as to the most appropriate indicators to be used, it does appear that such questions could feasibly be included as part of the standard DHS questionnaire.

One inherent issue with the use of standard DHS methodology however, is that it is reliant upon self-reporting for all variables related to pregnancy and childbirth, with a recall period of up to five years. In addition to the known variability of recall bias in general[46], validation studies comparing self-reported coverage of MNCH indicators to either health care records [47] or direct observation [48]suggest that the sensitivity and specificity of self-reported coverage can vary substantially, both between indicators and between contexts. Care should thus be taken in interpreting the QI, especially with regards to the potential for social desirability and recall bias to affect self-reporting of actions taken during the antenatal, delivery and postpartum period.

Similarly, while provision of emergency obstetric care can have a large impact on both maternal and neonatal mortality rates [49, 50], survivorship bias precludes the DHS from providing reliable measures relating to the treatment of potentially fatal conditions. Additionally, the variable weights produced by the PCA process do not reflect the relative importance of any given intervention in preventing death or disability. As a result, we can only consider the QI to be a partial indicator of true quality of maternal and neonatal care–however given the lack of regularly available measures in the existing literature even this imperfect measure may provide significant benefits to our understanding of quality of care in LMICs. Ideally, these findings will stimulate further research into the inclusion of a more diverse range of quality indicators in standard DHS based surveys. With further refinements, it is possible that the QI might be used to compare quality between countries, and provide an additional tool for researchers and policymakers to investigate of the effect of different health system elements on quality of care.

## Conclusions

As demonstrated using the Indonesia 2012 DHS, the Quality Index provides a method through which data collected as part of routine DHS programs may be used to examine disparities in the quality of maternal and neonatal health care in LMICs. The resulting analysis can provide important insights into both the current state of quality of care and the potential avenues for health system intervention. In Indonesia, for example, the analysis noted particular disparities in the quality of care received by the poor as well as those living in outlying provinces. The QI thus represents an important step forward in efforts to understand and improve quality of MNCH care in developing countries. It allows policymakers and development partners to measure, track progress and compare the quality of MNCH care across countries and within target populations.

## Supporting Information

S1 File Potential Indicators and Rationale for Use. (DOCX)

S2 File Sensitivity Analyses. (DOCX)
