# Supplementary material for: Measuring Quality of Maternal and Newborn Care in Developing Countries Using Demographic and Health Surveys
Source: PLoS One. 2016 Jun 30;11(6):e0157110. doi: 10.1371/journal.pone.0157110 (PMC4928810; doi:10.1371/journal.pone.0157110)
Supplement: S1 File — (DOCX) [file pone.0157110.s001.docx]

# S1: Potential Indicators and Rationale for Use

As the modules included in the DHS may be subject to country specific needs, the exact indicators used in each country’s analysis may differ. The following sections detail the indicators included in the core DHS 6 questionnaire, those included in HIV and Malaria endemic areas, and additional indicators that have been included in recent DHS not covered in the standard questionnaire. A summary of the potential indicators are provided in Tables A1.1, A1.2 and A1.3, with the following subsection providing a rationale for each indicators use.

## Core Indicators

There are thirteen potential quality indicators available in the core DHS questionnaire, seven relating to ANC and six relating to birth and delivery care.

**Table S1.1:** Potential Quality Indicators based on Core DHS questionnaire

| **Indicator** | **DHS recode VI variables** |
| --- | --- |
| At least 1 ANC visit in 1st Trimester | m14_1 (# of ANC visits)  m13_1 (Timing of 1st visit - months) |
| Blood Pressure measured during ANC | m42c_1 |
| Urine sample taken during ANC | m42d_1 |
| Blood sample taken during ANC | m42e_1 |
| 270+ days of Iron Supplementation during pregnancy | m45_1 (ever taken iron supplements during pregnancy) m46_1 (days of iron supplementation during pregnancy) |
| Fully protected from Tetanus during pregnancy | m1_1 (number of TT injections this pregnancy)  m1a_1 (number of TT injections prior to this pregnancy) |
| Told about pregnancy complications during ANC and where to seek help | m43_1 (Told about pregnancy complications)  m44_1 (Told where to go for complications) |
| Baby was weighed at birth | m19a_1 |
| Baby was breastfed within 1 hr of birth | m4_1 (Baby ever breastfed), m34_1 (Time after birth baby first breastfed) |
| No liquids given before milk began to flow (no prelacteal feed) | m4_1 (Baby ever breastfed), m55z_1 (First 3 days, given nothing (but breastmilk)) |
| Maternal postnatal check within 2 hrs of delivery | m50_1 (Mother received checkup after delivery), m51_1 (Timing of mother's checkup after delivery) |
| Neonatal postnatal check within 2 hrs of delivery | m70_1 (Baby received checkup after delivery), m71_1 (Timing of baby's checkup after delivery) |
| Mother received postpartum Vitamin A within 2 months of delivery | m54_1 |

The first potential indicator relates to the number and timing of ANC visits. According to IMPAC guidelines, pregnant women should ideally have a minimum of 4 ANC visits, starting with at least one visit in the first trimester, one in the second trimester, and at least two in the third trimester. As the total number of visits may better reflect coverage rather than quality, the chosen indicators instead represent the presence of absence of appropriately timed visits. However, in the core DHS questionnaire, timing of ANC visits is only asked in regards to the first ANC visit- the final indicator thus makes an assumption a correctly timed first visit is itself an indicator that additional visits also occurred at appropriate intervals.

The next set of indicators relate to the actions undertaken as part of the ANC process. In particular, the DHS asks about whether particular diagnostic tests were provided to the patient. These include whether or not the patient’s blood pressure was checked (used to screen issues related to high or low blood pressure), if a blood sample taken (to screen for various conditions such as anaemia and HIV) and if a urine sample taken (to screen for conditions such as pre-eclampsia and some STDs). These tests are indicative of specific provider actions that should be undertaken in every pregnancy as part of good quality care, regardless of the presence of absence of other maternal risk factors.

In addition to these diagnostic tests, the DHS also collects information about preventative care in the form of iron supplementation and tetanus immunisation during pregnancy. These questions were asked of all women regardless of whether or not they sought ANC, however they are a critical component of good quality ANC. Two more indicators are based on this set of questions.

According IMCPAC guidelines, all women should be routinely taking IFA once daily until 3 months post-delivery. The DHS asks if iron supplementation was taken during the pregnancy, and if so, for how many days was it taken. While the standard for “best quality” coverage according to IMPAC guidelines is 270 days or more of supplementation, other studies have used 180 days as a cut-off and it is possible there may be some effect of lower levels of quality. For this reason multiple levels of supplementation should be included in the analysis.

To prevent tetanus IMPAC recommends that a woman should receive at least 5 Tetanus Toxoid (TT) doses over a minimum 3 year period (3 in first year and one each in year 2 and 3). In practice, this means for women who have never received TT prior should receive at least 2 doses during their pregnancy, women with less than 5 doses in total should receive at least 1 dose during pregnancy and women with 5 doses do not need further immunisation. This definition does differ slightly from the standard DHS algorithm for determining tetanus protection, however for consistency the IMPAC definitions were used in the analysis. As with Iron supplementation, it is possible that there may be some effect of partial coverage, and thus multiple categories were included in the analysis.

ANC is considered a particularly important opportunity to advise expectant mothers on relevant issues that may arise as a result of their pregnancy, particularly in regards to their and their children’s health. The content covered as part of an ANC visit may vary depending on local conditions; however one of the most important issues to cover is potential warning signs that may indicate a problem with the pregnancy. The core DHS includes a two part question asking if women were told about potential signs of pregnancy complication, and if so, if they were advised about where to seek treatment. This forms the last of the core ANC indicators. While additional questions regarding advices during ANC would be beneficial, these questions at least provide an indicator that some form of health counselling was provided as part of ANC.

Unfortunately, no questions are included in the standard DHS questionnaire regarding actions taken during the delivery itself, although this may change in future revisions.^^[[1]](#footnote-1)^^ Instead, the indicators included here reflect actions taken immediately following the delivery.

The first birth related indictor relates to the child’s birth weight. Weighing the newborn to determine if it is low birth weight is an important step in determining the health of the baby after birth – low birth weight may be an indicator that additional supportive care is required. The DHS collects information about whether or not the baby was weighted at birth and it has been included as an indicator of quality care in the analysis as the identification of LBW is one of the key steps outlined in the IMPAC guidelines for immediate newborn care.

IMPAC guidelines also recommend that breastfeeding be initiated within one hour of delivery, and that no prelacteal feed^^[[2]](#footnote-2)^^ should be given in order to provide the maximum health benefit. While decisions regarding infant feeding ultimately rest with the mother, good quality care should include appropriate advice and support for breastfeeding. Inappropriate breastfeeding may be indicative that the support provided at the time of delivery was inadequate. It is for this reason that breastfeeding initiation and exclusivity for the first three days are included as the second and third birth related indicators.

The final set of indicators relate to the postnatal care received by mother and child. According to IMPAC both the woman and baby’s health should be monitored throughout the birth with the first (formal) examination occurring at least one hour post-delivery, with further check-ups until discharge (which should not be for at least 12 hours post-delivery). However the DHS only records the timing of the first reported health check - a mother who was checked immediately post-delivery as part of the birth monitoring may also have been checked more formally after the first hour. In terms of mortality, the most dangerous period of time is the first couple of hours following the birth. For this reason I have chosen to define “good quality” as having had a check-up within two hours of delivery. As the DHS collects information on both maternal and neonatal check-up I will treat these questions as two separate indicators.

Ideally both a maternal and a neonatal check should have occurred within the first two hours, however as with other quantitative indicators additional categories were included in the analysis representing lower levels of quality. The categories used were: check-up 3-12 hours post-delivery, check-up 13-24 hours post-delivery, and check-up 49 or more hours post-delivery. The same categories were be applied to both maternal and neonatal indicators

The last indicator for postnatal care is whether or not the mother was provided with a postpartum dose of vitamin A. This should be given either soon after delivery as a preventative measure to support maternal health during the postpartum period. There is no information regarding the timing of the dose in the DHS, only whether or not it was given within two months of delivery.

## Disease related Indicators

In addition to the core DHS questionnaire, in countries with a high HIV or Malaria prevalence, additional modules are included covering programs designed to address these diseases as part of ANC and delivery care. Six potential disease specific indicators were identified, one related to malaria prevention during pregnancy, four related to HIV testing and knowledge and one related to treatment for intestinal parasites.

**Table S1.2:** Potential Quality Indicators based on additional modules in DHS questionnaire

| **Indicator** | **DHS recode VI variables** |
| --- | --- |
| Received IPTp during pregnancy to prevent malaria | m49a_1 (During pregnancy took SP/Fansidar for malaria) - m49y_1 (took no drug for malaria) |
| Offered AIDS test prior to delivery | v839 (Offered AIDS test during ANC)  v839a (Offered HIV test between time went for delivery and before baby was born) |
| Advised about AIDS transmission from mother to child during ANC | v838a |
| Advised about things to do to prevent AIDS during ANC | v838b |
| Advised about getting tested for AIDS virus during ANC | v838c |
| Took drugs for intestinal parasites during pregnancy | m60_1 |

In areas of high malaria transmission it is recommended that during pregnancy women receive intermittent preventative therapy (IPT) for malaria. The appropriate regimen may vary depending on the species of malaria present and the level of drug resistance in the area. Thus good quality ANC should include a locally appropriate regimen for malaria treatment and prevention.

In countries with a high prevalence of HIV, it is recommended that all women be offered voluntary counselling and testing (VCT) regarding HIV during ANC. This initially involves the provision of advice about the transmission of HIV (particularly from mother to child), advice about prevention of HIV and advice about the need for HIV testing. Good quality ANC should involve counselling on all these topics. HIV testing should also be offered as part of good quality ANC as early detection will allow for the timely initiation of PMTCT (Prevention of Maternal to Child Transmission) if it is required. Women who are not tested as part of ANC should be offered a test when they arrive for delivery – this will be treated as a category of lower quality.

While deworming is technically included in the core DHS questionnaire, the process is not a standard part of ANC in all countries, and has been excluded from at least one eligible survey.^^[[3]](#footnote-3)^^ For this reason this indicator is included in the disease related category of indicators, as when it is present it is another indicator of appropriate ANC.

## Country specific Indicators

For countries who wish to examine particular health issues not otherwise covered by existing DHS modules, additional questions may be inserted into the questionnaire. As these questions may be specific to only one DHS, their inclusion must be considered on a case by case basis. The following section explores the use of questions included specifically in the Indonesia 2012 DHS as quality indicators, providing an example of what types of questions may be available.

**Table S1.3:** Potential Quality Indicators Specific to Indonesia 2012 DHS questionnaire

| **Indicator** | **DHS recode VI variables** |
| --- | --- |
| At least 1 ANC visit in 2nd Trimester | s412bb_01 (# of ANC visits in 2nd Trimester) |
| At least 2 ANC visits in 3rd Trimester | s412bc_01 (# of ANC visits in 3rd Trimester) |
| Weight measured during ANC | m42a_1 |
| Height measured during ANC | m42b_1 |
| Stomach examined during ANC | s413f_01 |
| Consultation during ANC | s413g_01 |
| Received MNCH book during ANC | s409b_01 |
| Discussed place of delivery during pregnancy | s414ba_01 |
| Discussed transportation to place of delivery during pregnancy | s414bb_01 |
| Discussed who would assist delivery during pregnancy | s414bc_01 |
| Discussed payment for delivery during pregnancy | s414bd_01 |
| Discussed possible blood donor during pregnancy | s414be_01 |

In addition to questions regarding the initiation of ANC, the Indonesia 2012 DHS also included questions about the number of ANC visits occurring in each trimester. As a result, two additional indicators (at least one visit in 2^nd^ trimester; at least two visits in 3^rd^ trimester) may be included in the analysis. An additional category of “1 visit in 3^rd^ trimester” may also be included as a lower quality measure.

In addition to asking questions about blood pressure, urine and blood testing, the Indonesia 2012 DHS included questions about whether the patient’s weight and height were measured, if the stomach was examined, if a consultation^^[[4]](#footnote-4)^^ was given and if the patient was provided with a “MNCH book” to keep track of health visits. These actions represent specific aspects of ANC considered by the Indonesian government to be representative of national guidelines regarding good quality care..

Another Indonesia specific set of indicators is the set of questions regarding birth preparedness. These questions ask if the respondent discussed issues such as place of delivery, transportation, birth assistance, payment for delivery and blood donation with anyone during her pregnancy. Ideally, these issues should be brought up as part of ANC advice and discussed with both the health provider and immediate family. If the woman does not report having discussed these issues, then she has not received the best possible ANC care. For this reason these questions as indicators are included.

1. While some DHS may carry information about sterile birth practices and temperature control, the inclusion of these questions are non-standard and they are rarely asked of facility deliveries [↑](#footnote-ref-1)
2. This denotes the provision of non-colostrum liquids such as water or sugar water within the first three days following birth, before breast milk starts to flow regularly. [↑](#footnote-ref-2)
3. Indonesia 2012 DHS [↑](#footnote-ref-3)
4. The definition of consultation used in the questionnaire is somewhat vague. I have assumed, based on contextual factors, that it represents a one-on-one discussion with a provider regarding the pregnancy. [↑](#footnote-ref-4)
