# Supplementary material for: Measuring Quality of Maternal and Newborn Care in Developing Countries Using Demographic and Health Surveys
Source: PLoS One. 2016 Jun 30;11(6):e0157110. doi: 10.1371/journal.pone.0157110 (PMC4928810; doi:10.1371/journal.pone.0157110)
Supplement: S2 File — (DOCX) [file pone.0157110.s002.docx]

# S2: Sensitivity Analyses

## Inclusion of Observations with Missing Data.

To test the assumptions used to minimise the impact of missing observations, parts of the analysis were redone using a dataset in which all observations that had any missing data, or “don’t know” responses were dropped prior to the construction of the PCA process. Table B1.1 summarises the variable weights derived using two indicator sets on each dataset. As can be seen, the omission of the observations with missing data has minimal impact on the resulting variable weights. Given the importance of these observations to maintaining the representativeness of the sample, a decision was made to include these observations in the final analysis.

**Table S2.1:** PCA Variable weights using imputed and non-missing datasets

|  |  |  | Missing data imputed:  All Indicators, <5 categories +prelacteal feeding | Missing data imputed:  DHS Indicators, <5 categories +prelacteal feeding | Missing data dropped:  All Indicators, <5 categories +prelacteal feeding | Missing data dropped:  DHS Indicators, <5 categories +prelacteal feeding |
| --- | --- | --- | --- | --- | --- | --- |
| ANC visit in 1st Trimester | | | 0.167 | 0.193 | 0.173 | 0.199 |
| ANC visit in 2nd Trimester | | | 0.152 |  | 0.146 |  |
| ANC visits in 3rd Trimester | 1 | | -0.012 |  | -0.013 |  |
|  | 2 | | 0.188 |  | 0.183 |  |
|  | None | | -0.176 |  | -0.170 |  |
| Weight measured during ANC | | | 0.172 |  | 0.177 |  |
| Height measured during ANC | | | 0.190 |  | 0.189 |  |
| Blood Pressure measured during ANC | | | 0.156 | 0.162 | 0.158 | 0.164 |
| Urine sample taken during ANC | | | 0.202 | 0.263 | 0.203 | 0.265 |
| Blood sample taken during ANC | | | 0.171 | 0.220 | 0.174 | 0.222 |
| Stomach examined during ANC | | | 0.122 |  | 0.125 |  |
| Consultation during ANC | | | 0.194 |  | 0.196 |  |
| Received MNCH book during ANC | | | 0.199 |  | 0.201 |  |
| Iron Supplementation during pregnancy | Full (270+ days) | | 0.025 | 0.031 | 0.010 | 0.033 |
|  | Partial | 1-29 days | 0.009 | 0.012 | 0.055 | 0.012 |
|  |  | 30-89 days | 0.065 | 0.088 | 0.053 | 0.073 |
|  |  | 90-179 days | 0.049 | 0.061 | 0.057 | 0.067 |
|  |  | 180-269 days | 0.054 | 0.066 | 0.027 | 0.071 |
|  | None | | -0.201 | -0.257 | -0.201 | -0.256 |
| Tetanus Immunisation | Full Protection | | 0.206 | 0.285 | 0.207 | 0.283 |
|  | Partial Protection | | -0.020 | -0.047 | -0.021 | -0.047 |
|  | None | | -0.186 | -0.238 | -0.185 | -0.236 |
| Pregnancy complication Advice | Symptoms only | | 0.000 | 0.000 | 0.000 | 0.001 |
|  | Symptoms and Help | | 0.270 | 0.396 | 0.268 | 0.388 |
|  | None | | -0.270 | -0.396 | -0.269 | -0.389 |
| Discussed place of delivery during pregnancy | | | 0.216 |  | 0.212 |  |
| Discussed transportation to place of delivery during pregnancy | | | 0.255 |  | 0.253 |  |
| Discussed who would assist delivery during pregnancy | | | 0.204 |  | 0.202 |  |
| Discussed payment for delivery during pregnancy | | | 0.214 |  | 0.210 |  |
| Discussed possible blood donor during pregnancy | | | 0.107 |  | 0.109 |  |
| Baby was weighed at birth | | | 0.156 | 0.177 | 0.154 | 0.175 |
| Baby was breastfed within 1 hr of birth | | | 0.029 | 0.049 | 0.037 | 0.059 |
| No liquids given before milk began to flow (no prelacteal feed) | | | -0.023 | -0.010 | -0.018 | -0.004 |
| Maternal postnatal check | Full (<2hrs) | | 0.147 | 0.218 | 0.152 | 0.226 |
|  | Partial | 3-12 hrs | 0.025 | 0.011 | 0.024 | 0.010 |
|  |  | 13-24hrs | -0.005 | -0.018 | -0.005 | -0.020 |
|  |  | 25-48hrs | -0.004 | -0.007 | -0.005 | -0.007 |
|  |  | 49hrs + | -0.012 | -0.020 | -0.013 | -0.021 |
|  | None | | -0.151 | -0.184 | -0.153 | -0.188 |
| Neonatal postnatal check | Full (<2hrs) | | 0.140 | 0.215 | 0.143 | 0.220 |
|  | Partial | 3-12 hrs | 0.018 | 0.017 | 0.019 | 0.018 |
|  |  | 13-24hrs | 0.007 | 0.005 | 0.007 | 0.005 |
|  |  | 25-48hrs | 0.001 | 0.002 | 0.002 | 0.002 |
|  |  | 49hrs + | 0.011 | 0.017 | 0.011 | 0.015 |
|  | None | | -0.178 | -0.256 | -0.181 | -0.260 |
| Postpartum Vitamin A within 2 months of delivery | | | 0.155 | 0.233 | 0.157 | 0.234 |
| **Rho** |  |  | **0.1752** | **0.1589** | **0.1776** | **0.1613** |

## Consistency of PCA derived weights

To test the consistency in PCA derived weights, half of the sample was randomly selected and each PCA index was recalculated based upon that subsample. This process of random selection and recalculation was carried out a total of ten times for each index, with Table B2.1 showing the minimum and maximum weights obtained through the process, and variation in mean, minimum and maximum scores in Table B2.2. In generally, the range of each index, as well as the individual variable weights, are remarkably similar across the subsamples. This suggests that while there is individual variation, the underlying correlations identified by the PCA process are fairly static across the sample as a whole.

**Table S2.2:** Variation in PCA derived weights based on random sub-samples

| Indicators | | | Scenario 1 | | Scenario 2 | | Scenario 3 | | Scenario 4 | | Scenario 5 | | Scenario 6 | | Scenario 7 | | Scenario 8 | |
| --- | --- | --- | --- | --- | --- | --- | --- | --- | --- | --- | --- | --- | --- | --- | --- | --- | --- | --- |
|  |  |  | All Indicators, <5 categories +prelacteal feeding | | DHS Indicators, <5 categories +prelacteal feeding | | All Indicators, <5 categories | | DHS Indicators, <5 categories | | All Indicators, <3 categories | | DHS Indicators, <3 categories | | All indicators, < 3 categories - Any iron supplementation | | DHS indicators, < 3 categories - Any iron supplementation | |
|  |  |  | MIN | MAX | MIN | MAX | MIN | MAX | MIN | MAX | MIN | MAX | MIN | MAX | MIN | MAX | MIN | MAX |
| ANC visit in 1st Trimester | | | 0.163 | 0.172 | 0.188 | 0.197 | 0.160 | 0.173 | 0.181 | 0.204 | 0.157 | 0.168 | 0.179 | 0.189 | 0.163 | 0.172 | 0.179 | 0.204 |
| ANC visit in 2nd Trimester | | | 0.144 | 0.159 |  |  | 0.150 | 0.152 |  |  | 0.146 | 0.154 |  |  | 0.144 | 0.159 |  |  |
| ANC visits in 3rd Trimester | 1 |  | -0.012 | -0.011 |  |  | -0.015 | -0.008 |  |  | -0.015 | -0.008 |  |  | -0.012 | -0.011 |  |  |
|  | 2 |  | 0.181 | 0.194 |  |  | 0.185 | 0.190 |  |  | 0.182 | 0.189 |  |  | 0.181 | 0.195 |  |  |
|  | None |  | -0.182 | -0.169 |  |  | -0.179 | -0.172 |  |  | -0.177 | -0.170 |  |  | -0.182 | -0.170 |  |  |
| Weight measured during ANC | | | 0.16 | 0.176 |  |  | 0.169 | 0.174 |  |  | 0.166 | 0.174 |  |  | 0.167 | 0.177 |  |  |
| Height measured during ANC | | | 0.18 | 0.192 |  |  | 0.184 | 0.195 |  |  | 0.180 | 0.191 |  |  | 0.189 | 0.193 |  |  |
| Blood Pressure measured during ANC | | | 0.149 | 0.161 | 0.154 | 0.169 | 0.150 | 0.160 | 0.155 | 0.167 | 0.150 | 0.158 | 0.153 | 0.167 | 0.150 | 0.161 | 0.153 | 0.166 |
| Urine sample taken during ANC | | | 0.199 | 0.203 | 0.255 | 0.271 | 0.196 | 0.206 | 0.259 | 0.267 | 0.190 | 0.201 | 0.240 | 0.255 | 0.200 | 0.205 | 0.261 | 0.269 |
| Blood sample taken during ANC | | | 0.160 | 0.180 | 0.206 | 0.233 | 0.164 | 0.177 | 0.210 | 0.23 | 0.158 | 0.173 | 0.194 | 0.218 | 0.161 | 0.182 | 0.213 | 0.233 |
| Stomach examined during ANC | | | 0.120 | 0.124 |  |  | 0.116 | 0.128 |  |  | 0.118 | 0.124 |  |  | 0.120 | 0.125 |  |  |
| Consultation during ANC | | | 0.190 | 0.197 |  |  | 0.186 | 0.200 |  |  | 0.188 | 0.192 |  |  | 0.191 | 0.198 |  |  |
| Received MNCH book during ANC | | | 0.193 | 0.205 |  |  | 0.194 | 0.203 |  |  | 0.194 | 0.199 |  |  | 0.194 | 0.205 |  |  |
| Iron Supplementation during pregnancy | Full (270+ days) | | 0.006 | 0.012 | 0.009 | 0.014 | 0.002 | 0.015 | 0.001 | 0.021 | 0.017 | 0.021 | 0.016 | 0.021 | 0.190 | 0.202 | 0.236 | 0.251 |
|  | Partial (1-269 days) | 1-29 days | 0.061 | 0.067 | 0.082 | 0.092 | 0.06 | 0.067 | 0.081 | 0.093 | 0.196 | 0.203 | 0.271 | 0.285 |  |  |  |  |
|  |  | 30-89 days | 0.044 | 0.05 | 0.056 | 0.066 | 0.044 | 0.053 | 0.054 | 0.068 |  |  |  |  |  |  |  |  |
|  |  | 90-179 days | 0.051 | 0.056 | 0.062 | 0.069 | 0.051 | 0.055 | 0.063 | 0.068 |  |  |  |  |  |  |  |  |
|  |  | 180-269 days | 0.023 | 0.027 | 0.027 | 0.033 | 0.023 | 0.027 | 0.028 | 0.033 |  |  |  |  |  |  |  |  |
|  | None |  | -0.207 | -0.195 | -0.264 | -0.249 | -0.206 | -0.195 | -0.264 | -0.248 | -0.222 | -0.216 | -0.304 | -0.290 |  |  |  |  |
| Tetanus Immunisation | Full Protection | | 0.199 | 0.213 | 0.277 | 0.291 | 0.200 | 0.212 | 0.274 | 0.294 | 0.197 | 0.213 | 0.271 | 0.286 | 0.200 | 0.214 | 0.274 | 0.293 |
|  | Partial Protection | | -0.024 | -0.016 | -0.053 | -0.040 | -0.027 | -0.013 | -0.055 | -0.037 | -0.025 | -0.014 | -0.050 | -0.036 | -0.024 | -0.016 | -0.056 | -0.037 |
|  | None |  | -0.192 | -0.179 | -0.246 | -0.229 | -0.191 | -0.181 | -0.242 | -0.234 | -0.191 | -0.179 | -0.243 | -0.225 | -0.193 | -0.180 | -0.241 | -0.233 |
| Pregnancy complication Advice | Symptoms only | | -0.001 | 0.001 | -0.001 | 0.001 | -0.001 | 0.001 | -0.001 | 0.001 | -0.001 | 0.001 | -0.002 | 0.002 | -0.001 | 0.001 | -0.001 | 0.001 |
|  | Symptoms and Help | | 0.262 | 0.277 | 0.387 | 0.403 | 0.266 | 0.273 | 0.389 | 0.401 | 0.260 | 0.264 | 0.362 | 0.376 | 0.264 | 0.280 | 0.393 | 0.407 |
|  | None |  | -0.276 | -0.263 | -0.402 | -0.389 | -0.273 | -0.266 | -0.401 | -0.389 | -0.264 | -0.260 | -0.375 | -0.362 | -0.279 | -0.265 | -0.407 | -0.393 |
| Discussed place of delivery during pregnancy | | | 0.212 | 0.220 |  |  | 0.209 | 0.222 |  |  | 0.205 | 0.214 |  |  | 0.214 | 0.221 |  |  |
| Discussed transportation to place of delivery during pregnancy | | | 0.249 | 0.260 |  |  | 0.248 | 0.261 |  |  | 0.242 | 0.249 |  |  | 0.251 | 0.262 |  |  |
| Discussed who would assist delivery during pregnancy | | | 0.199 | 0.208 |  |  | 0.199 | 0.208 |  |  | 0.194 | 0.201 |  |  | 0.201 | 0.210 |  |  |
| Discussed payment for delivery during pregnancy | | | 0.210 | 0.217 |  |  | 0.210 | 0.216 |  |  | 0.201 | 0.212 |  |  | 0.211 | 0.218 |  |  |
| Discussed possible blood donor during pregnancy | | | 0.101 | 0.112 |  |  | 0.105 | 0.108 |  |  | 0.098 | 0.108 |  |  | 0.102 | 0.113 |  |  |
| Baby was weighed at birth | | | 0.150 | 0.161 | 0.171 | 0.182 | 0.151 | 0.159 | 0.172 | 0.180 | 0.149 | 0.155 | 0.165 | 0.174 | 0.150 | 0.161 | 0.172 | 0.178 |
| Baby was breastfed within 1 hr of birth | | | 0.023 | 0.034 | 0.040 | 0.057 | 0.024 | 0.038 | 0.041 | 0.060 | 0.022 | 0.036 | 0.041 | 0.052 | 0.025 | 0.036 | 0.042 | 0.062 |
| No liquids given before milk began to flow (no prelacteal feed) | | | -0.029 | -0.015 | -0.023 | 0.002 |  |  |  |  |  |  |  |  |  |  |  |  |
| Maternal postnatal check | Full (<2hrs) | | 0.137 | 0.155 | 0.201 | 0.233 | 0.141 | 0.153 | 0.207 | 0.229 | 0.134 | 0.145 | 0.182 | 0.224 | 0.135 | 0.155 | 0.217 | 0.249 |
|  | Partial (3+ hrs) | 3-12 hrs | 0.021 | 0.027 | 0.006 | 0.015 | 0.021 | 0.026 | 0.006 | 0.014 | 0.004 | 0.012 | -0.038 | -0.015 | 0.001 | 0.009 | -0.071 | -0.031 |
|  |  | 13-24hrs | -0.007 | -0.001 | -0.023 | -0.012 | -0.008 | -0.001 | -0.026 | -0.010 |  |  |  |  |  |  |  |  |
|  |  | 25-48hrs | -0.006 | -0.001 | -0.01 | -0.003 | -0.005 | -0.003 | -0.008 | -0.005 |  |  |  |  |  |  |  |  |
|  |  | 49hrs + | -0.014 | -0.009 | -0.022 | -0.017 | -0.014 | -0.009 | -0.024 | -0.015 |  |  |  |  |  |  |  |  |
|  | None |  | -0.156 | -0.144 | -0.193 | -0.174 | -0.156 | -0.145 | -0.189 | -0.178 | -0.154 | -0.142 | -0.185 | -0.167 | -0.157 | -0.144 | -0.186 | -0.177 |
| Neonatal postnatal check | Full (<2hrs) | | 0.13 | 0.14 | 0.207 | 0.221 | 0.136 | 0.143 | 0.204 | 0.224 | 0.129 | 0.136 | 0.189 | 0.206 | 0.131 | 0.142 | 0.204 | 0.233 |
|  | Partial (3+ hrs) | 3-12 hrs | 0.014 | 0.021 | 0.011 | 0.021 | 0.016 | 0.019 | 0.013 | 0.019 | 0.039 | 0.051 | 0.044 | 0.063 | 0.037 | 0.050 | 0.024 | 0.058 |
|  |  | 13-24hrs | 0.004 | 0.009 | 0.002 | 0.008 | 0.004 | 0.008 | 0.003 | 0.007 |  |  |  |  |  |  |  |  |
|  |  | 25-48hrs | 0.000 | 0.002 | 0.000 | 0.002 | -0.000 | 0.002 | -0.001 | 0.004 |  |  |  |  |  |  |  |  |
|  |  | 49hrs + | 0.005 | 0.016 | 0.011 | 0.021 | 0.007 | 0.015 | 0.011 | 0.022 |  |  |  |  |  |  |  |  |
|  | None |  | -0.18 | -0.174 | -0.259 | -0.251 | -0.181 | -0.173 | -0.264 | -0.246 | -0.186 | -0.170 | -0.255 | -0.248 | -0.183 | -0.178 | -0.271 | -0.250 |
| Postpartum Vitamin A within 2 months of delivery | | | 0.151 | 0.158 | 0.228 | 0.237 | 0.146 | 0.163 | 0.223 | 0.242 | 0.149 | 0.158 | 0.219 | 0.230 | 0.152 | 0.160 | 0.225 | 0.244 |
|  |  |  |  |  |  |  |  |  |  |  |  |  |  |  |  |  |  |  |
| Rho |  |  | 0.1715 | 0.1791 | 0.1561 | 0.1619 | 0.1772 | 0.1856 | 0.1639 | 0.1701 | 0.197 | 0.2066 | 0.1935 | 0.2004 | 0.1994 | 0.2078 | 0.1941 | 0.2017 |

**Table S2.3:** Variation in Mean, Minimum and Max scores for random subsamples

|  | Mean |  | Lowest Score | | Highest Score | |
| --- | --- | --- | --- | --- | --- | --- |
|  | MIN | MAX | MIN | MAX | MIN | MAX |
| Scenario 1 | -1.16E-09 | 1.16E-09 | -3.182 | -3.145 | 1.607 | 1.627 |
| Scenario 2 | -1.13E-09 | 1.07E-09 | -2.542 | -2.517 | 1.804 | 1.832 |
| Scenario 3 | -1.29E-09 | 1.50E-09 | -3.160 | -3.145 | 1.607 | 1.617 |
| Scenario 4 | -2.28E-09 | 2.67E-09 | -2.526 | -2.517 | 1.807 | 1.824 |
| Scenario 5 | -1.01E-09 | 5.44E-10 | -3.192 | -3.162 | 1.566 | 1.580 |
| Scenario 6 | -1.62E-09 | 3.05E-09 | -2.594 | -2.562 | 1.713 | 1.758 |
| Scenario 7 | -1.26E-09 | 1.12E-09 | -3.183 | -3.140 | 1.578 | 1.593 |
| Scenario 8 | -2.95E-09 | 1.23E-09 | -2.502 | -2.474 | 1.777 | 1.829 |

## Mean Indicator Values by Quintile for Each Scenario

If an index is to act as a compound indicator of quality of care, we would assume that those classified as having high quality by the index should also score highly according to individual quality indicators. The following tables provide the mean value for each quality indicator based on quintiles formed from the QI (Q1 being the lowest and Q5 being the highest) for each scenario. Both Equal Weight (EW) and PCA derived (PCA) index classification are included in the tables.

**Table S2.4:** Mean Indicator Values by Quintile – Scenario 1 All Indicators, <5 categories +prelacteal feeding

| Indicator |  |  | EW Quintile | |  |  |  | PCA Quintile | |  |  |  |
| --- | --- | --- | --- | --- | --- | --- | --- | --- | --- | --- | --- | --- |
|  |  |  | Q1 | Q2 | Q3 | Q4 | Q5 | Q1 | Q2 | Q3 | Q4 | Q5 |
| ANC visit in 1st Trimester |  |  | 0.436 | 0.741 | 0.817 | 0.865 | 0.942 | 0.443 | 0.734 | 0.809 | 0.864 | 0.944 |
| ANC visit in 2nd Trimester |  |  | 0.621 | 0.931 | 0.963 | 0.988 | 0.996 | 0.587 | 0.939 | 0.978 | 0.992 | 0.999 |
| ANC visits in 3rd Trimester | 1 |  | 0.037 | 0.006 | 0.002 | 0.001 | 0 | 0.044 | 0.003 | 0 | 0 | 0 |
|  | 2 |  | 0.508 | 0.863 | 0.926 | 0.957 | 0.983 | 0.428 | 0.863 | 0.952 | 0.987 | 0.999 |
|  | None |  | 0.455 | 0.131 | 0.072 | 0.042 | 0.017 | 0.528 | 0.134 | 0.048 | 0.013 | 0.001 |
| Weight measured during ANC |  |  | 0.555 | 0.936 | 0.985 | 0.995 | 1 | 0.538 | 0.945 | 0.987 | 0.997 | 1 |
| Height measured during ANC |  |  | 0.127 | 0.301 | 0.422 | 0.575 | 0.785 | 0.154 | 0.345 | 0.422 | 0.532 | 0.744 |
| Blood Pressure measured during ANC |  |  | 0.616 | 0.955 | 0.983 | 0.995 | 0.999 | 0.6 | 0.96 | 0.987 | 0.998 | 0.999 |
| Urine sample taken during ANC |  |  | 0.092 | 0.257 | 0.378 | 0.552 | 0.801 | 0.117 | 0.279 | 0.383 | 0.521 | 0.765 |
| Blood sample taken during ANC |  |  | 0.127 | 0.255 | 0.327 | 0.492 | 0.769 | 0.159 | 0.286 | 0.356 | 0.464 | 0.695 |
| Stomach examined during ANC |  |  | 0.726 | 0.98 | 0.994 | 0.993 | 0.999 | 0.718 | 0.979 | 0.994 | 0.998 | 1 |
| Consultation during ANC |  |  | 0.422 | 0.78 | 0.871 | 0.921 | 0.969 | 0.421 | 0.766 | 0.86 | 0.929 | 0.98 |
| Received MNCH book during ANC |  |  | 0.395 | 0.759 | 0.858 | 0.912 | 0.959 | 0.401 | 0.754 | 0.84 | 0.913 | 0.965 |
| Iron Supplementation during pregnancy | Full (270+ days) |  | 0.003 | 0.026 | 0.04 | 0.061 | 0.112 | 0.004 | 0.026 | 0.038 | 0.071 | 0.101 |
|  | Partial (1-269 days) | 1-29 days | 0.207 | 0.251 | 0.217 | 0.194 | 0.151 | 0.183 | 0.244 | 0.233 | 0.205 | 0.156 |
|  |  | 30-89 days | 0.151 | 0.277 | 0.291 | 0.284 | 0.273 | 0.129 | 0.264 | 0.284 | 0.308 | 0.289 |
|  |  | 90-179 days | 0.038 | 0.098 | 0.132 | 0.155 | 0.182 | 0.031 | 0.096 | 0.124 | 0.148 | 0.202 |
|  |  | 180-269 days | 0.016 | 0.053 | 0.104 | 0.145 | 0.192 | 0.013 | 0.047 | 0.096 | 0.139 | 0.212 |
|  | None |  | 0.585 | 0.295 | 0.215 | 0.161 | 0.091 | 0.639 | 0.324 | 0.224 | 0.129 | 0.039 |
| Tetanus Immunisation | Full Protection | | 0.298 | 0.558 | 0.662 | 0.736 | 0.832 | 0.267 | 0.537 | 0.645 | 0.743 | 0.884 |
|  | Partial Protection | | 0.175 | 0.21 | 0.181 | 0.165 | 0.116 | 0.176 | 0.22 | 0.199 | 0.161 | 0.094 |
|  | None |  | 0.528 | 0.231 | 0.157 | 0.099 | 0.051 | 0.557 | 0.243 | 0.156 | 0.096 | 0.022 |
| Pregnancy complication Advice | Symptoms only | | 0.019 | 0.037 | 0.027 | 0.021 | 0.014 | 0.017 | 0.036 | 0.028 | 0.025 | 0.012 |
|  | Symptoms and Help | | 0.098 | 0.318 | 0.474 | 0.643 | 0.806 | 0.096 | 0.269 | 0.429 | 0.628 | 0.903 |
|  | None |  | 0.883 | 0.644 | 0.499 | 0.336 | 0.18 | 0.887 | 0.695 | 0.543 | 0.347 | 0.085 |
| Discussed place of delivery during pregnancy |  |  | 0.358 | 0.714 | 0.917 | 0.968 | 0.996 | 0.386 | 0.682 | 0.898 | 0.976 | 0.997 |
| Discussed transportation to place of delivery during pregnancy | | | 0.114 | 0.396 | 0.656 | 0.81 | 0.932 | 0.165 | 0.382 | 0.616 | 0.797 | 0.929 |
| Discussed who would assist delivery during pregnancy | |  | 0.39 | 0.704 | 0.896 | 0.961 | 0.994 | 0.424 | 0.668 | 0.873 | 0.972 | 0.995 |
| Discussed payment for delivery during pregnancy |  |  | 0.333 | 0.656 | 0.845 | 0.919 | 0.976 | 0.364 | 0.626 | 0.821 | 0.927 | 0.978 |
| Discussed possible blood donor during pregnancy | |  | 0.016 | 0.044 | 0.096 | 0.189 | 0.458 | 0.024 | 0.059 | 0.109 | 0.184 | 0.42 |
| Baby was weighed at birth |  |  | 0.56 | 0.884 | 0.946 | 0.975 | 0.992 | 0.547 | 0.871 | 0.954 | 0.981 | 0.998 |
| Baby was breastfed within 1 hr of birth |  |  | 0.36 | 0.38 | 0.436 | 0.531 | 0.712 | 0.446 | 0.457 | 0.461 | 0.499 | 0.549 |
| No liquids given before milk began to flow (no prelacteal feed) | | | 0.349 | 0.295 | 0.311 | 0.374 | 0.568 | 0.429 | 0.374 | 0.347 | 0.361 | 0.382 |
| Maternal postnatal check | Full (<2hrs) | | 0.233 | 0.402 | 0.484 | 0.575 | 0.68 | 0.24 | 0.405 | 0.489 | 0.543 | 0.689 |
|  | Partial (3+ hrs) | 3-12 hrs | 0.111 | 0.186 | 0.209 | 0.212 | 0.18 | 0.103 | 0.179 | 0.21 | 0.223 | 0.181 |
|  |  | 13-24hrs | 0.116 | 0.139 | 0.127 | 0.11 | 0.079 | 0.102 | 0.135 | 0.134 | 0.121 | 0.079 |
|  |  | 25-48hrs | 0.03 | 0.032 | 0.023 | 0.015 | 0.012 | 0.027 | 0.035 | 0.02 | 0.022 | 0.009 |
|  |  | 49hrs + | 0.106 | 0.103 | 0.089 | 0.054 | 0.035 | 0.092 | 0.104 | 0.089 | 0.066 | 0.039 |
|  | None |  | 0.403 | 0.138 | 0.068 | 0.035 | 0.015 | 0.436 | 0.144 | 0.057 | 0.025 | 0.003 |
| Neonatal postnatal check | Full (<2hrs) | | 0.097 | 0.188 | 0.275 | 0.373 | 0.539 | 0.099 | 0.186 | 0.275 | 0.359 | 0.545 |
|  | Partial (3+ hrs) | 3-12 hrs | 0.051 | 0.092 | 0.107 | 0.114 | 0.114 | 0.049 | 0.09 | 0.1 | 0.128 | 0.109 |
|  |  | 13-24hrs | 0.059 | 0.084 | 0.082 | 0.083 | 0.066 | 0.052 | 0.08 | 0.086 | 0.086 | 0.069 |
|  |  | 25-48hrs | 0.017 | 0.02 | 0.023 | 0.018 | 0.015 | 0.013 | 0.023 | 0.021 | 0.02 | 0.016 |
|  |  | 49hrs + | 0.158 | 0.189 | 0.18 | 0.173 | 0.126 | 0.144 | 0.183 | 0.168 | 0.171 | 0.161 |
|  | None |  | 0.618 | 0.427 | 0.333 | 0.24 | 0.141 | 0.643 | 0.439 | 0.35 | 0.236 | 0.1 |
| Postpartum Vitamin A within 2 months of delivery | |  | 0.211 | 0.348 | 0.446 | 0.581 | 0.736 | 0.238 | 0.378 | 0.447 | 0.55 | 0.698 |

**Table S2.5:** Mean Indicator Values by Quintile – Scenario 2 DHS Indicators, <5 categories +prelacteal feeding

| Indicator |  |  | EW Quintile | |  | |  | |  | | PCA Quintile | |  | |  | |  | |
| --- | --- | --- | --- | --- | --- | --- | --- | --- | --- | --- | --- | --- | --- | --- | --- | --- | --- | --- |
|  |  |  | Q1 | Q2 | | Q3 | | Q4 | | Q5 | Q1 | Q2 | | Q3 | | Q4 | | Q5 |
| ANC visit in 1st Trimester |  |  | 0.404 | 0.737 | | 0.833 | | 0.889 | | 0.948 | 0.462 | 0.747 | | 0.811 | | 0.853 | | 0.921 |
| ANC visit in 2nd Trimester |  |  |  |  | |  | |  | |  |  |  | |  | |  | |  |
| ANC visits in 3rd Trimester | 1 |  |  |  | |  | |  | |  |  |  | |  | |  | |  |
|  | 2 |  |  |  | |  | |  | |  |  |  | |  | |  | |  |
|  | None |  |  |  | |  | |  | |  |  |  | |  | |  | |  |
| Weight measured during ANC |  |  |  |  | |  | |  | |  |  |  | |  | |  | |  |
| Height measured during ANC |  |  |  |  | |  | |  | |  |  |  | |  | |  | |  |
| Blood Pressure measured during ANC |  |  | 0.626 | 0.947 | | 0.984 | | 0.993 | | 0.998 | 0.634 | 0.952 | | 0.973 | | 0.988 | | 0.996 |
| Urine sample taken during ANC |  |  | 0.062 | 0.241 | | 0.405 | | 0.585 | | 0.812 | 0.100 | 0.267 | | 0.403 | | 0.537 | | 0.759 |
| Blood sample taken during ANC |  |  | 0.094 | 0.236 | | 0.377 | | 0.526 | | 0.760 | 0.125 | 0.284 | | 0.395 | | 0.484 | | 0.671 |
| Stomach examined during ANC |  |  |  |  | |  | |  | |  |  |  | |  | |  | |  |
| Consultation during ANC |  |  |  |  | |  | |  | |  |  |  | |  | |  | |  |
| Received MNCH book during ANC |  |  |  |  | |  | |  | |  |  |  | |  | |  | |  |
| Iron Supplementation during pregnancy | Full (270+ days) | | 0.003 | 0.020 | | 0.046 | | 0.060 | | 0.115 | 0.006 | 0.032 | | 0.057 | | 0.062 | | 0.084 |
|  | Partial (1-269 days) | 1-29 days | 0.213 | 0.241 | | 0.244 | | 0.177 | | 0.140 | 0.162 | 0.254 | | 0.214 | | 0.218 | | 0.173 |
|  |  | 30-89 days | 0.130 | 0.281 | | 0.283 | | 0.337 | | 0.248 | 0.099 | 0.251 | | 0.296 | | 0.303 | | 0.324 |
|  |  | 90-179 days | 0.031 | 0.093 | | 0.142 | | 0.143 | | 0.201 | 0.033 | 0.096 | | 0.132 | | 0.152 | | 0.189 |
|  |  | 180-269 days | 0.011 | 0.064 | | 0.080 | | 0.141 | | 0.219 | 0.018 | 0.072 | | 0.099 | | 0.128 | | 0.191 |
|  | None |  | 0.612 | 0.301 | | 0.205 | | 0.142 | | 0.076 | 0.681 | 0.295 | | 0.204 | | 0.138 | | 0.038 |
| Tetanus Immunisation | Full Protection | | 0.265 | 0.557 | | 0.679 | | 0.744 | | 0.854 | 0.188 | 0.549 | | 0.673 | | 0.743 | | 0.925 |
|  | Partial Protection | | 0.180 | 0.206 | | 0.182 | | 0.172 | | 0.105 | 0.208 | 0.233 | | 0.188 | | 0.162 | | 0.059 |
|  | None |  | 0.555 | 0.236 | | 0.140 | | 0.084 | | 0.041 | 0.604 | 0.218 | | 0.139 | | 0.095 | | 0.016 |
| Pregnancy complication Advice | Symptoms only | | 0.018 | 0.033 | | 0.026 | | 0.027 | | 0.014 | 0.014 | 0.031 | | 0.037 | | 0.026 | | 0.011 |
|  | Symptoms and Help | | 0.094 | 0.330 | | 0.502 | | 0.632 | | 0.802 | 0.019 | 0.131 | | 0.408 | | 0.777 | | 0.989 |
|  | None |  | 0.888 | 0.638 | | 0.473 | | 0.341 | | 0.183 | 0.967 | 0.838 | | 0.555 | | 0.197 | | 0.000 |
| Discussed place of delivery during pregnancy |  |  |  |  | |  | |  | |  |  |  | |  | |  | |  |
| Discussed transportation to place of delivery during pregnancy | | |  |  | |  | |  | |  |  |  | |  | |  | |  |
| Discussed who would assist delivery during pregnancy | |  |  |  | |  | |  | |  |  |  | |  | |  | |  |
| Discussed payment for delivery during pregnancy |  |  |  |  | |  | |  | |  |  |  | |  | |  | |  |
| Discussed possible blood donor during pregnancy | |  |  |  | |  | |  | |  |  |  | |  | |  | |  |
| Baby was weighed at birth |  |  | 0.553 | 0.890 | | 0.948 | | 0.977 | | 0.993 | 0.580 | 0.880 | | 0.937 | | 0.964 | | 0.990 |
| Baby was breastfed within 1 hr of birth |  |  | 0.288 | 0.337 | | 0.457 | | 0.572 | | 0.787 | 0.428 | 0.440 | | 0.477 | | 0.501 | | 0.566 |
| No liquids given before milk began to flow (no prelacteal feed) | | | 0.273 | 0.255 | | 0.319 | | 0.417 | | 0.649 | 0.408 | 0.357 | | 0.353 | | 0.374 | | 0.400 |
| Maternal postnatal check | Full (<2hrs) | | 0.212 | 0.390 | | 0.483 | | 0.604 | | 0.701 | 0.203 | 0.387 | | 0.484 | | 0.553 | | 0.739 |
|  | Partial (3+ hrs) | 3-12 hrs | 0.107 | 0.197 | | 0.213 | | 0.205 | | 0.175 | 0.121 | 0.209 | | 0.216 | | 0.205 | | 0.144 |
|  |  | 13-24hrs | 0.112 | 0.150 | | 0.133 | | 0.091 | | 0.079 | 0.117 | 0.147 | | 0.130 | | 0.115 | | 0.062 |
|  |  | 25-48hrs | 0.033 | 0.030 | | 0.023 | | 0.013 | | 0.011 | 0.030 | 0.029 | | 0.023 | | 0.018 | | 0.012 |
|  |  | 49hrs + | 0.114 | 0.107 | | 0.085 | | 0.052 | | 0.025 | 0.090 | 0.106 | | 0.082 | | 0.073 | | 0.038 |
|  | None |  | 0.422 | 0.126 | | 0.062 | | 0.036 | | 0.008 | 0.438 | 0.121 | | 0.066 | | 0.035 | | 0.005 |
| Neonatal postnatal check | Full (<2hrs) | | 0.073 | 0.171 | | 0.273 | | 0.397 | | 0.574 | 0.051 | 0.170 | | 0.297 | | 0.353 | | 0.591 |
|  | Partial (3+ hrs) | 3-12 hrs | 0.042 | 0.092 | | 0.115 | | 0.118 | | 0.112 | 0.049 | 0.107 | | 0.110 | | 0.117 | | 0.093 |
|  |  | 13-24hrs | 0.054 | 0.086 | | 0.085 | | 0.078 | | 0.071 | 0.051 | 0.090 | | 0.086 | | 0.083 | | 0.063 |
|  |  | 25-48hrs | 0.014 | 0.024 | | 0.023 | | 0.016 | | 0.015 | 0.015 | 0.018 | | 0.021 | | 0.022 | | 0.016 |
|  |  | 49hrs + | 0.157 | 0.190 | | 0.195 | | 0.167 | | 0.115 | 0.121 | 0.172 | | 0.180 | | 0.188 | | 0.164 |
|  | None |  | 0.660 | 0.438 | | 0.308 | | 0.224 | | 0.114 | 0.711 | 0.442 | | 0.306 | | 0.237 | | 0.072 |
| Postpartum Vitamin A within 2 months of delivery | |  | 0.134 | 0.329 | | 0.485 | | 0.612 | | 0.785 | 0.164 | 0.362 | | 0.477 | | 0.572 | | 0.737 |

**Table S2.6:** Mean Indicator Values by Quintile – Scenario 3 All Indicators, <5 categories

| Indicator |  |  | EW Quintile | |  | |  | |  | | PCA Quintile | |  | |  | |  | |
| --- | --- | --- | --- | --- | --- | --- | --- | --- | --- | --- | --- | --- | --- | --- | --- | --- | --- | --- |
|  |  |  | Q1 | Q2 | | Q3 | | Q4 | | Q5 | Q1 | Q2 | | Q3 | | Q4 | | Q5 |
| ANC visit in 1st Trimester |  |  | 0.435 | 0.734 | | 0.813 | | 0.873 | | 0.946 | 0.454 | 0.746 | | 0.810 | | 0.855 | | 0.927 |
| ANC visit in 2nd Trimester |  |  | 0.619 | 0.930 | | 0.968 | | 0.987 | | 0.995 | 0.621 | 0.930 | | 0.969 | | 0.980 | | 0.995 |
| ANC visits in 3rd Trimester | 1 |  | 0.038 | 0.005 | | 0.002 | | 0.001 | | 0.000 | 0.038 | 0.006 | | 0.002 | | 0.002 | | 0.000 |
|  | 2 |  | 0.506 | 0.864 | | 0.922 | | 0.960 | | 0.984 | 0.487 | 0.861 | | 0.933 | | 0.959 | | 0.989 |
|  | None |  | 0.456 | 0.131 | | 0.076 | | 0.039 | | 0.016 | 0.475 | 0.133 | | 0.065 | | 0.040 | | 0.011 |
| Weight measured during ANC |  |  | 0.553 | 0.940 | | 0.982 | | 0.996 | | 1.000 | 0.562 | 0.935 | | 0.978 | | 0.992 | | 0.999 |
| Height measured during ANC |  |  | 0.127 | 0.312 | | 0.403 | | 0.572 | | 0.798 | 0.136 | 0.326 | | 0.419 | | 0.555 | | 0.762 |
| Blood Pressure measured during ANC |  |  | 0.613 | 0.957 | | 0.983 | | 0.997 | | 0.998 | 0.616 | 0.958 | | 0.980 | | 0.992 | | 0.998 |
| Urine sample taken during ANC |  |  | 0.089 | 0.258 | | 0.370 | | 0.546 | | 0.818 | 0.103 | 0.273 | | 0.382 | | 0.529 | | 0.779 |
| Blood sample taken during ANC |  |  | 0.121 | 0.267 | | 0.325 | | 0.482 | | 0.779 | 0.141 | 0.272 | | 0.360 | | 0.474 | | 0.713 |
| Stomach examined during ANC |  |  | 0.726 | 0.981 | | 0.991 | | 0.996 | | 0.999 | 0.728 | 0.978 | | 0.991 | | 0.995 | | 0.997 |
| Consultation during ANC |  |  | 0.416 | 0.778 | | 0.868 | | 0.928 | | 0.974 | 0.428 | 0.775 | | 0.862 | | 0.922 | | 0.968 |
| Received MNCH book during ANC |  |  | 0.397 | 0.753 | | 0.858 | | 0.911 | | 0.963 | 0.398 | 0.762 | | 0.851 | | 0.902 | | 0.960 |
| Iron Supplementation during pregnancy | Full (270+ days) | | 0.004 | 0.024 | | 0.037 | | 0.064 | | 0.114 | 0.004 | 0.034 | | 0.047 | | 0.066 | | 0.088 |
|  | Partial (1-269 days) | 1-29 days | 0.205 | 0.251 | | 0.234 | | 0.186 | | 0.142 | 0.184 | 0.231 | | 0.225 | | 0.211 | | 0.170 |
|  |  | 30-89 days | 0.157 | 0.280 | | 0.294 | | 0.291 | | 0.251 | 0.129 | 0.261 | | 0.289 | | 0.294 | | 0.301 |
|  |  | 90-179 days | 0.035 | 0.091 | | 0.128 | | 0.151 | | 0.200 | 0.033 | 0.098 | | 0.128 | | 0.150 | | 0.193 |
|  |  | 180-269 days | 0.015 | 0.048 | | 0.092 | | 0.141 | | 0.215 | 0.018 | 0.057 | | 0.097 | | 0.145 | | 0.191 |
|  | None |  | 0.584 | 0.305 | | 0.213 | | 0.167 | | 0.078 | 0.631 | 0.319 | | 0.214 | | 0.135 | | 0.056 |
| Tetanus Immunisation | Full Protection | | 0.303 | 0.562 | | 0.660 | | 0.727 | | 0.835 | 0.257 | 0.534 | | 0.659 | | 0.741 | | 0.886 |
|  | Partial Protection | | 0.166 | 0.208 | | 0.188 | | 0.168 | | 0.116 | 0.182 | 0.228 | | 0.192 | | 0.161 | | 0.086 |
|  | None |  | 0.531 | 0.231 | | 0.152 | | 0.104 | | 0.048 | 0.561 | 0.238 | | 0.149 | | 0.098 | | 0.028 |
| Pregnancy complication Advice | Symptoms only | | 0.017 | 0.040 | | 0.023 | | 0.024 | | 0.013 | 0.013 | 0.041 | | 0.031 | | 0.022 | | 0.012 |
|  | Symptoms and Help | | 0.093 | 0.312 | | 0.470 | | 0.646 | | 0.818 | 0.054 | 0.218 | | 0.415 | | 0.702 | | 0.936 |
|  | None |  | 0.890 | 0.648 | | 0.507 | | 0.329 | | 0.168 | 0.933 | 0.741 | | 0.554 | | 0.276 | | 0.052 |
| Discussed place of delivery during pregnancy |  |  | 0.348 | 0.714 | | 0.918 | | 0.974 | | 0.996 | 0.356 | 0.719 | | 0.904 | | 0.966 | | 0.995 |
| Discussed transportation to place of delivery during pregnancy | | | 0.115 | 0.385 | | 0.651 | | 0.815 | | 0.941 | 0.125 | 0.398 | | 0.641 | | 0.792 | | 0.933 |
| Discussed who would assist delivery during pregnancy | |  | 0.381 | 0.700 | | 0.898 | | 0.967 | | 0.995 | 0.394 | 0.704 | | 0.881 | | 0.959 | | 0.994 |
| Discussed payment for delivery during pregnancy |  |  | 0.326 | 0.647 | | 0.848 | | 0.927 | | 0.979 | 0.336 | 0.657 | | 0.830 | | 0.918 | | 0.975 |
| Discussed possible blood donor during pregnancy | |  | 0.015 | 0.039 | | 0.098 | | 0.190 | | 0.465 | 0.017 | 0.055 | | 0.120 | | 0.205 | | 0.398 |
| Baby was weighed at birth |  |  | 0.557 | 0.878 | | 0.951 | | 0.977 | | 0.995 | 0.570 | 0.872 | | 0.947 | | 0.972 | | 0.991 |
| Baby was breastfed within 1 hr of birth |  |  | 0.390 | 0.395 | | 0.442 | | 0.528 | | 0.666 | 0.430 | 0.458 | | 0.462 | | 0.495 | | 0.569 |
| No liquids given before milk began to flow (no prelacteal feed) | | |  |  | |  | |  | |  |  |  | |  | |  | |  |
| Maternal postnatal check | Full (<2hrs) | | 0.240 | 0.410 | | 0.481 | | 0.557 | | 0.688 | 0.226 | 0.405 | | 0.477 | | 0.552 | | 0.706 |
|  | Partial (3+ hrs) | 3-12 hrs | 0.109 | 0.177 | | 0.214 | | 0.218 | | 0.178 | 0.119 | 0.187 | | 0.220 | | 0.213 | | 0.157 |
|  |  | 13-24hrs | 0.109 | 0.137 | | 0.133 | | 0.112 | | 0.079 | 0.112 | 0.142 | | 0.128 | | 0.115 | | 0.074 |
|  |  | 25-48hrs | 0.031 | 0.030 | | 0.023 | | 0.019 | | 0.010 | 0.030 | 0.030 | | 0.024 | | 0.017 | | 0.013 |
|  |  | 49hrs + | 0.104 | 0.102 | | 0.088 | | 0.061 | | 0.033 | 0.093 | 0.098 | | 0.090 | | 0.067 | | 0.042 |
|  | None |  | 0.407 | 0.145 | | 0.061 | | 0.034 | | 0.013 | 0.419 | 0.139 | | 0.061 | | 0.037 | | 0.009 |
| Neonatal postnatal check | Full (<2hrs) | | 0.098 | 0.191 | | 0.267 | | 0.368 | | 0.549 | 0.086 | 0.181 | | 0.277 | | 0.365 | | 0.553 |
|  | Partial (3+ hrs) | 3-12 hrs | 0.050 | 0.083 | | 0.110 | | 0.118 | | 0.116 | 0.056 | 0.093 | | 0.108 | | 0.124 | | 0.095 |
|  |  | 13-24hrs | 0.056 | 0.081 | | 0.083 | | 0.086 | | 0.068 | 0.054 | 0.083 | | 0.083 | | 0.089 | | 0.064 |
|  |  | 25-48hrs | 0.016 | 0.021 | | 0.022 | | 0.018 | | 0.015 | 0.014 | 0.022 | | 0.020 | | 0.019 | | 0.017 |
|  |  | 49hrs + | 0.157 | 0.189 | | 0.183 | | 0.172 | | 0.123 | 0.140 | 0.176 | | 0.174 | | 0.171 | | 0.167 |
|  | None |  | 0.623 | 0.435 | | 0.334 | | 0.237 | | 0.129 | 0.649 | 0.445 | | 0.338 | | 0.231 | | 0.105 |
| Postpartum Vitamin A within 2 months of delivery | |  | 0.214 | 0.340 | | 0.450 | | 0.575 | | 0.745 | 0.225 | 0.354 | | 0.463 | | 0.553 | | 0.718 |

**Table S2.7:** Mean Indicator Values by Quintile – Scenario 4 DHS Indicators, <5 categories

| Indicator |  |  | EW Quintile | |  | |  | |  | | PCA Quintile | |  | |  | |  | |
| --- | --- | --- | --- | --- | --- | --- | --- | --- | --- | --- | --- | --- | --- | --- | --- | --- | --- | --- |
|  |  |  | Q1 | Q2 | | Q3 | | Q4 | | Q5 | Q1 | Q2 | | Q3 | | Q4 | | Q5 |
| ANC visit in 1st Trimester |  |  | 0.396 | 0.724 | | 0.839 | | 0.887 | | 0.962 | 0.462 | 0.746 | | 0.812 | | 0.851 | | 0.921 |
| ANC visit in 2nd Trimester |  |  |  |  | |  | |  | |  |  |  | |  | |  | |  |
| ANC visits in 3rd Trimester | 1 |  |  |  | |  | |  | |  |  |  | |  | |  | |  |
|  | 2 |  |  |  | |  | |  | |  |  |  | |  | |  | |  |
|  | None |  |  |  | |  | |  | |  |  |  | |  | |  | |  |
| Weight measured during ANC |  |  |  |  | |  | |  | |  |  |  | |  | |  | |  |
| Height measured during ANC |  |  |  |  | |  | |  | |  |  |  | |  | |  | |  |
| Blood Pressure measured during ANC |  |  | 0.619 | 0.953 | | 0.987 | | 0.994 | | 0.999 | 0.635 | 0.952 | | 0.973 | | 0.988 | | 0.996 |
| Urine sample taken during ANC |  |  | 0.057 | 0.217 | | 0.386 | | 0.584 | | 0.840 | 0.101 | 0.267 | | 0.402 | | 0.536 | | 0.760 |
| Blood sample taken during ANC |  |  | 0.091 | 0.230 | | 0.349 | | 0.530 | | 0.775 | 0.125 | 0.284 | | 0.395 | | 0.483 | | 0.673 |
| Stomach examined during ANC |  |  |  |  | |  | |  | |  |  |  | |  | |  | |  |
| Consultation during ANC |  |  |  |  | |  | |  | |  |  |  | |  | |  | |  |
| Received MNCH book during ANC |  |  |  |  | |  | |  | |  |  |  | |  | |  | |  |
| Iron Supplementation during pregnancy | Full (270+ days) | | 0.003 | 0.015 | | 0.041 | | 0.069 | | 0.115 | 0.006 | 0.031 | | 0.056 | | 0.062 | | 0.084 |
|  | Partial (1-269 days) | 1-29 days | 0.196 | 0.263 | | 0.230 | | 0.177 | | 0.154 | 0.161 | 0.255 | | 0.215 | | 0.216 | | 0.173 |
|  |  | 30-89 days | 0.132 | 0.290 | | 0.288 | | 0.293 | | 0.274 | 0.099 | 0.252 | | 0.296 | | 0.301 | | 0.327 |
|  |  | 90-179 days | 0.035 | 0.094 | | 0.139 | | 0.143 | | 0.194 | 0.033 | 0.097 | | 0.131 | | 0.152 | | 0.189 |
|  |  | 180-269 days | 0.015 | 0.053 | | 0.100 | | 0.133 | | 0.212 | 0.017 | 0.072 | | 0.099 | | 0.131 | | 0.189 |
|  | None |  | 0.619 | 0.285 | | 0.203 | | 0.185 | | 0.051 | 0.683 | 0.293 | | 0.203 | | 0.138 | | 0.038 |
| Tetanus Immunisation | Full Protection | | 0.259 | 0.567 | | 0.654 | | 0.753 | | 0.857 | 0.187 | 0.548 | | 0.674 | | 0.744 | | 0.923 |
|  | Partial Protection | | 0.189 | 0.197 | | 0.196 | | 0.160 | | 0.106 | 0.208 | 0.233 | | 0.187 | | 0.161 | | 0.060 |
|  | None |  | 0.552 | 0.235 | | 0.150 | | 0.087 | | 0.037 | 0.604 | 0.219 | | 0.139 | | 0.095 | | 0.017 |
| Pregnancy complication Advice | Symptoms only | | 0.019 | 0.029 | | 0.031 | | 0.026 | | 0.014 | 0.014 | 0.031 | | 0.037 | | 0.026 | | 0.011 |
|  | Symptoms and Help | | 0.094 | 0.291 | | 0.493 | | 0.638 | | 0.828 | 0.019 | 0.133 | | 0.408 | | 0.776 | | 0.989 |
|  | None |  | 0.886 | 0.680 | | 0.476 | | 0.336 | | 0.159 | 0.967 | 0.837 | | 0.556 | | 0.197 | | 0.000 |
| Discussed place of delivery during pregnancy |  |  |  |  | |  | |  | |  |  |  | |  | |  | |  |
| Discussed transportation to place of delivery during pregnancy | | |  |  | |  | |  | |  |  |  | |  | |  | |  |
| Discussed who would assist delivery during pregnancy | |  |  |  | |  | |  | |  |  |  | |  | |  | |  |
| Discussed payment for delivery during pregnancy |  |  |  |  | |  | |  | |  |  |  | |  | |  | |  |
| Discussed possible blood donor during pregnancy | |  |  |  | |  | |  | |  |  |  | |  | |  | |  |
| Baby was weighed at birth |  |  | 0.541 | 0.888 | | 0.956 | | 0.982 | | 0.995 | 0.581 | 0.880 | | 0.937 | | 0.963 | | 0.990 |
| Baby was breastfed within 1 hr of birth |  |  | 0.327 | 0.369 | | 0.451 | | 0.546 | | 0.729 | 0.424 | 0.439 | | 0.479 | | 0.502 | | 0.569 |
| No liquids given before milk began to flow (no prelacteal feed) | | |  |  | |  | |  | |  |  |  | |  | |  | |  |
| Maternal postnatal check | Full (<2hrs) | | 0.205 | 0.389 | | 0.475 | | 0.596 | | 0.714 | 0.202 | 0.388 | | 0.483 | | 0.554 | | 0.739 |
|  | Partial (3+ hrs) | 3-12 hrs | 0.099 | 0.189 | | 0.226 | | 0.203 | | 0.182 | 0.122 | 0.211 | | 0.215 | | 0.204 | | 0.144 |
|  |  | 13-24hrs | 0.112 | 0.149 | | 0.133 | | 0.107 | | 0.070 | 0.119 | 0.145 | | 0.131 | | 0.114 | | 0.062 |
|  |  | 25-48hrs | 0.034 | 0.029 | | 0.025 | | 0.017 | | 0.007 | 0.030 | 0.030 | | 0.023 | | 0.018 | | 0.012 |
|  |  | 49hrs + | 0.118 | 0.115 | | 0.085 | | 0.047 | | 0.023 | 0.090 | 0.105 | | 0.082 | | 0.074 | | 0.038 |
|  | None |  | 0.433 | 0.130 | | 0.057 | | 0.030 | | 0.005 | 0.437 | 0.122 | | 0.066 | | 0.036 | | 0.005 |
| Neonatal postnatal check | Full (<2hrs) | | 0.067 | 0.169 | | 0.260 | | 0.379 | | 0.599 | 0.053 | 0.169 | | 0.298 | | 0.353 | | 0.591 |
|  | Partial (3+ hrs) | 3-12 hrs | 0.041 | 0.079 | | 0.117 | | 0.122 | | 0.120 | 0.050 | 0.107 | | 0.109 | | 0.118 | | 0.093 |
|  |  | 13-24hrs | 0.050 | 0.087 | | 0.088 | | 0.090 | | 0.060 | 0.052 | 0.088 | | 0.086 | | 0.084 | | 0.063 |
|  |  | 25-48hrs | 0.016 | 0.020 | | 0.024 | | 0.019 | | 0.013 | 0.015 | 0.018 | | 0.021 | | 0.022 | | 0.016 |
|  |  | 49hrs + | 0.158 | 0.202 | | 0.192 | | 0.163 | | 0.110 | 0.122 | 0.174 | | 0.178 | | 0.190 | | 0.163 |
|  | None |  | 0.668 | 0.442 | | 0.318 | | 0.227 | | 0.098 | 0.708 | 0.444 | | 0.307 | | 0.234 | | 0.074 |
| Postpartum Vitamin A within 2 months of delivery | |  | 0.137 | 0.318 | | 0.462 | | 0.617 | | 0.795 | 0.163 | 0.361 | | 0.479 | | 0.571 | | 0.738 |

**Table S2.8:** Mean Indicator Values by Quintile – Scenario 5 All Indicators, 3 categories

| Indicator |  |  | EW Quintile | |  | |  | |  | | PCA Quintile | |  | |  | |  | |
| --- | --- | --- | --- | --- | --- | --- | --- | --- | --- | --- | --- | --- | --- | --- | --- | --- | --- | --- |
|  |  |  | Q1 | Q2 | | Q3 | | Q4 | | Q5 | Q1 | Q2 | | Q3 | | Q4 | | Q5 |
| ANC visit in 1st Trimester |  |  | 0.443 | 0.742 | | 0.823 | | 0.880 | | 0.955 | 0.459 | 0.744 | | 0.808 | | 0.861 | | 0.921 |
| ANC visit in 2nd Trimester |  |  | 0.630 | 0.934 | | 0.971 | | 0.988 | | 0.996 | 0.619 | 0.932 | | 0.967 | | 0.984 | | 0.993 |
| ANC visits in 3rd Trimester | 1 |  | 0.037 | 0.005 | | 0.002 | | 0.001 | | 0.000 | 0.038 | 0.007 | | 0.001 | | 0.001 | | 0.000 |
|  | 2 |  | 0.519 | 0.872 | | 0.925 | | 0.963 | | 0.986 | 0.485 | 0.865 | | 0.932 | | 0.960 | | 0.987 |
|  | None |  | 0.444 | 0.123 | | 0.072 | | 0.036 | | 0.014 | 0.478 | 0.128 | | 0.066 | | 0.039 | | 0.013 |
| Weight measured during ANC |  |  | 0.568 | 0.944 | | 0.985 | | 0.996 | | 1.000 | 0.562 | 0.935 | | 0.979 | | 0.992 | | 0.999 |
| Height measured during ANC |  |  | 0.133 | 0.313 | | 0.426 | | 0.597 | | 0.823 | 0.141 | 0.323 | | 0.421 | | 0.558 | | 0.754 |
| Blood Pressure measured during ANC |  |  | 0.627 | 0.957 | | 0.987 | | 0.996 | | 0.999 | 0.618 | 0.956 | | 0.980 | | 0.991 | | 0.998 |
| Urine sample taken during ANC |  |  | 0.094 | 0.254 | | 0.400 | | 0.579 | | 0.845 | 0.107 | 0.275 | | 0.385 | | 0.518 | | 0.780 |
| Blood sample taken during ANC |  |  | 0.130 | 0.263 | | 0.336 | | 0.521 | | 0.812 | 0.142 | 0.280 | | 0.359 | | 0.469 | | 0.710 |
| Stomach examined during ANC |  |  | 0.736 | 0.981 | | 0.992 | | 0.997 | | 0.999 | 0.728 | 0.978 | | 0.991 | | 0.995 | | 0.997 |
| Consultation during ANC |  |  | 0.423 | 0.791 | | 0.879 | | 0.932 | | 0.976 | 0.438 | 0.770 | | 0.857 | | 0.926 | | 0.965 |
| Received MNCH book during ANC |  |  | 0.407 | 0.763 | | 0.867 | | 0.921 | | 0.964 | 0.395 | 0.768 | | 0.848 | | 0.902 | | 0.961 |
| Iron Supplementation during pregnancy | Full (270+ days) | | 0.004 | 0.026 | | 0.039 | | 0.068 | | 0.120 | 0.006 | 0.041 | | 0.054 | | 0.068 | | 0.070 |
|  | Partial (1-269 days) | 1-29 days | 0.414 | 0.679 | | 0.760 | | 0.782 | | 0.801 | 0.314 | 0.626 | | 0.746 | | 0.816 | | 0.903 |
|  |  | 30-89 days |  |  | |  | |  | |  |  |  | |  | |  | |  |
|  |  | 90-179 days |  |  | |  | |  | |  |  |  | |  | |  | |  |
|  |  | 180-269 days |  |  | |  | |  | |  |  |  | |  | |  | |  |
|  | None |  | 0.582 | 0.295 | | 0.201 | | 0.150 | | 0.080 | 0.680 | 0.332 | | 0.199 | | 0.116 | | 0.028 |
| Tetanus Immunisation | Full Protection | | 0.310 | 0.565 | | 0.673 | | 0.744 | | 0.843 | 0.256 | 0.530 | | 0.661 | | 0.740 | | 0.889 |
|  | Partial Protection | | 0.173 | 0.209 | | 0.183 | | 0.156 | | 0.116 | 0.182 | 0.231 | | 0.194 | | 0.159 | | 0.083 |
|  | None |  | 0.517 | 0.226 | | 0.145 | | 0.100 | | 0.042 | 0.562 | 0.238 | | 0.145 | | 0.101 | | 0.028 |
| Pregnancy complication Advice | Symptoms only | | 0.018 | 0.040 | | 0.023 | | 0.020 | | 0.014 | 0.015 | 0.038 | | 0.031 | | 0.020 | | 0.014 |
|  | Symptoms and Help | | 0.095 | 0.325 | | 0.494 | | 0.666 | | 0.841 | 0.058 | 0.220 | | 0.416 | | 0.701 | | 0.929 |
|  | None |  | 0.887 | 0.635 | | 0.483 | | 0.314 | | 0.145 | 0.927 | 0.742 | | 0.552 | | 0.279 | | 0.057 |
| Discussed place of delivery during pregnancy |  |  | 0.356 | 0.733 | | 0.929 | | 0.981 | | 0.998 | 0.369 | 0.715 | | 0.898 | | 0.964 | | 0.994 |
| Discussed transportation to place of delivery during pregnancy | | | 0.118 | 0.413 | | 0.663 | | 0.843 | | 0.952 | 0.141 | 0.399 | | 0.639 | | 0.786 | | 0.925 |
| Discussed who would assist delivery during pregnancy | |  | 0.385 | 0.722 | | 0.910 | | 0.975 | | 0.996 | 0.409 | 0.701 | | 0.871 | | 0.957 | | 0.993 |
| Discussed payment for delivery during pregnancy |  |  | 0.330 | 0.668 | | 0.858 | | 0.943 | | 0.980 | 0.353 | 0.653 | | 0.822 | | 0.918 | | 0.971 |
| Discussed possible blood donor during pregnancy | |  | 0.015 | 0.041 | | 0.107 | | 0.211 | | 0.503 | 0.021 | 0.055 | | 0.123 | | 0.200 | | 0.396 |
| Baby was weighed at birth |  |  | 0.567 | 0.888 | | 0.955 | | 0.978 | | 0.997 | 0.569 | 0.879 | | 0.943 | | 0.969 | | 0.991 |
| Baby was breastfed within 1 hr of birth |  |  | 0.389 | 0.396 | | 0.452 | | 0.543 | | 0.682 | 0.432 | 0.456 | | 0.466 | | 0.498 | | 0.561 |
| No liquids given before milk began to flow (no prelacteal feed) | | |  |  | |  | |  | |  |  |  | |  | |  | |  |
| Maternal postnatal check | Full (<2hrs) | | 0.241 | 0.402 | | 0.487 | | 0.572 | | 0.727 | 0.234 | 0.407 | | 0.485 | | 0.544 | | 0.695 |
|  | Partial (3+ hrs) | 3-12 hrs | 0.362 | 0.462 | | 0.457 | | 0.395 | | 0.262 | 0.342 | 0.461 | | 0.452 | | 0.418 | | 0.295 |
|  |  | 13-24hrs |  |  | |  | |  | |  |  |  | |  | |  | |  |
|  |  | 25-48hrs |  |  | |  | |  | |  |  |  | |  | |  | |  |
|  |  | 49hrs + |  |  | |  | |  | |  |  |  | |  | |  | |  |
|  | None |  | 0.397 | 0.135 | | 0.056 | | 0.033 | | 0.012 | 0.424 | 0.131 | | 0.063 | | 0.037 | | 0.009 |
| Neonatal postnatal check | Full (<2hrs) | | 0.101 | 0.192 | | 0.282 | | 0.389 | | 0.566 | 0.090 | 0.185 | | 0.284 | | 0.364 | | 0.540 |
|  | Partial (3+ hrs) | 3-12 hrs | 0.279 | 0.386 | | 0.393 | | 0.393 | | 0.309 | 0.250 | 0.369 | | 0.382 | | 0.406 | | 0.363 |
|  |  | 13-24hrs |  |  | |  | |  | |  |  |  | |  | |  | |  |
|  |  | 25-48hrs |  |  | |  | |  | |  |  |  | |  | |  | |  |
|  |  | 49hrs + |  |  | |  | |  | |  |  |  | |  | |  | |  |
|  | None |  | 0.620 | 0.422 | | 0.325 | | 0.219 | | 0.125 | 0.660 | 0.446 | | 0.334 | | 0.230 | | 0.097 |
| Postpartum Vitamin A within 2 months of delivery | |  | 0.217 | 0.347 | | 0.458 | | 0.596 | | 0.772 | 0.215 | 0.369 | | 0.451 | | 0.553 | | 0.723 |

**Table S2.9:** Mean Indicator Values by Quintile – Scenario 6 DHS Indicators, 3 categories

| Indicator |  |  | EW Quintile | |  | |  | |  | | PCA Quintile | |  | |  | |  | |
| --- | --- | --- | --- | --- | --- | --- | --- | --- | --- | --- | --- | --- | --- | --- | --- | --- | --- | --- |
|  |  |  | Q1 | Q2 | | Q3 | | Q4 | | Q5 | Q1 | Q2 | | Q3 | | Q4 | | Q5 |
| ANC visit in 1st Trimester |  |  | 0.401 | 0.733 | | 0.845 | | 0.903 | | 0.961 | 0.472 | 0.745 | | 0.806 | | 0.851 | | 0.921 |
| ANC visit in 2nd Trimester |  |  |  |  | |  | |  | |  |  |  | |  | |  | |  |
| ANC visits in 3rd Trimester | 1 |  |  |  | |  | |  | |  |  |  | |  | |  | |  |
|  | 2 |  |  |  | |  | |  | |  |  |  | |  | |  | |  |
|  | None |  |  |  | |  | |  | |  |  |  | |  | |  | |  |
| Weight measured during ANC |  |  |  |  | |  | |  | |  |  |  | |  | |  | |  |
| Height measured during ANC |  |  |  |  | |  | |  | |  |  |  | |  | |  | |  |
| Blood Pressure measured during ANC |  |  | 0.626 | 0.955 | | 0.987 | | 0.996 | | 0.999 | 0.637 | 0.947 | | 0.977 | | 0.986 | | 0.997 |
| Urine sample taken during ANC |  |  | 0.058 | 0.215 | | 0.426 | | 0.628 | | 0.858 | 0.109 | 0.270 | | 0.411 | | 0.534 | | 0.747 |
| Blood sample taken during ANC |  |  | 0.088 | 0.224 | | 0.388 | | 0.576 | | 0.791 | 0.135 | 0.291 | | 0.397 | | 0.473 | | 0.668 |
| Stomach examined during ANC |  |  |  |  | |  | |  | |  |  |  | |  | |  | |  |
| Consultation during ANC |  |  |  |  | |  | |  | |  |  |  | |  | |  | |  |
| Received MNCH book during ANC |  |  |  |  | |  | |  | |  |  |  | |  | |  | |  |
| Iron Supplementation during pregnancy | Full (270+ days) | | 0.003 | 0.016 | | 0.052 | | 0.067 | | 0.116 | 0.012 | 0.043 | | 0.061 | | 0.069 | | 0.054 |
|  | Partial (1-269 days) | 1-29 days | 0.371 | 0.711 | | 0.733 | | 0.796 | | 0.835 | 0.221 | 0.651 | | 0.757 | | 0.844 | | 0.934 |
|  |  | 30-89 days |  |  | |  | |  | |  |  |  | |  | |  | |  |
|  |  | 90-179 days |  |  | |  | |  | |  |  |  | |  | |  | |  |
|  |  | 180-269 days |  |  | |  | |  | |  |  |  | |  | |  | |  |
|  | None |  | 0.626 | 0.273 | | 0.215 | | 0.137 | | 0.049 | 0.767 | 0.305 | | 0.181 | | 0.086 | | 0.012 |
| Tetanus Immunisation | Full Protection | | 0.267 | 0.560 | | 0.667 | | 0.768 | | 0.885 | 0.192 | 0.531 | | 0.681 | | 0.754 | | 0.923 |
|  | Partial Protection | | 0.185 | 0.209 | | 0.198 | | 0.152 | | 0.083 | 0.207 | 0.241 | | 0.180 | | 0.159 | | 0.061 |
|  | None |  | 0.548 | 0.231 | | 0.136 | | 0.081 | | 0.033 | 0.601 | 0.228 | | 0.139 | | 0.087 | | 0.016 |
| Pregnancy complication Advice | Symptoms only | | 0.020 | 0.029 | | 0.029 | | 0.026 | | 0.012 | 0.015 | 0.034 | | 0.033 | | 0.026 | | 0.011 |
|  | Symptoms and Help | | 0.092 | 0.304 | | 0.515 | | 0.673 | | 0.840 | 0.037 | 0.171 | | 0.403 | | 0.730 | | 0.989 |
|  | None |  | 0.888 | 0.667 | | 0.456 | | 0.301 | | 0.148 | 0.948 | 0.795 | | 0.564 | | 0.245 | | 0.000 |
| Discussed place of delivery during pregnancy |  |  |  |  | |  | |  | |  |  |  | |  | |  | |  |
| Discussed transportation to place of delivery during pregnancy | | |  |  | |  | |  | |  |  |  | |  | |  | |  |
| Discussed who would assist delivery during pregnancy | |  |  |  | |  | |  | |  |  |  | |  | |  | |  |
| Discussed payment for delivery during pregnancy |  |  |  |  | |  | |  | |  |  |  | |  | |  | |  |
| Discussed possible blood donor during pregnancy | |  |  |  | |  | |  | |  |  |  | |  | |  | |  |
| Baby was weighed at birth |  |  | 0.551 | 0.892 | | 0.959 | | 0.984 | | 0.994 | 0.595 | 0.871 | | 0.935 | | 0.962 | | 0.990 |
| Baby was breastfed within 1 hr of birth |  |  | 0.325 | 0.362 | | 0.477 | | 0.568 | | 0.743 | 0.431 | 0.444 | | 0.474 | | 0.513 | | 0.552 |
| No liquids given before milk began to flow (no prelacteal feed) | | |  |  | |  | |  | |  |  |  | |  | |  | |  |
| Maternal postnatal check | Full (<2hrs) | | 0.201 | 0.376 | | 0.494 | | 0.609 | | 0.761 | 0.216 | 0.378 | | 0.479 | | 0.561 | | 0.735 |
|  | Partial (3+ hrs) | 3-12 hrs | 0.376 | 0.499 | | 0.451 | | 0.367 | | 0.234 | 0.369 | 0.484 | | 0.452 | | 0.405 | | 0.258 |
|  |  | 13-24hrs |  |  | |  | |  | |  |  |  | |  | |  | |  |
|  |  | 25-48hrs |  |  | |  | |  | |  |  |  | |  | |  | |  |
|  |  | 49hrs + |  |  | |  | |  | |  |  |  | |  | |  | |  |
|  | None |  | 0.423 | 0.125 | | 0.056 | | 0.025 | | 0.005 | 0.415 | 0.137 | | 0.069 | | 0.034 | | 0.007 |
| Neonatal postnatal check | Full (<2hrs) | | 0.068 | 0.171 | | 0.277 | | 0.403 | | 0.625 | 0.063 | 0.175 | | 0.281 | | 0.371 | | 0.577 |
|  | Partial (3+ hrs) | 3-12 hrs | 0.261 | 0.402 | | 0.412 | | 0.401 | | 0.282 | 0.238 | 0.368 | | 0.393 | | 0.422 | | 0.347 |
|  |  | 13-24hrs |  |  | |  | |  | |  |  |  | |  | |  | |  |
|  |  | 25-48hrs |  |  | |  | |  | |  |  |  | |  | |  | |  |
|  |  | 49hrs + |  |  | |  | |  | |  |  |  | |  | |  | |  |
|  | None |  | 0.671 | 0.427 | | 0.310 | | 0.196 | | 0.093 | 0.699 | 0.457 | | 0.326 | | 0.206 | | 0.077 |
| Postpartum Vitamin A within 2 months of delivery | |  | 0.132 | 0.316 | | 0.493 | | 0.647 | | 0.819 | 0.172 | 0.341 | | 0.489 | | 0.579 | | 0.735 |

**Table S2.10:** Mean Indicator Values by Quintile – Scenario 7 All Indicators, 3 categories Any Iron

| Indicator |  |  | EW Quintile | |  | |  | |  | | PCA Quintile | |  | |  | |  | |
| --- | --- | --- | --- | --- | --- | --- | --- | --- | --- | --- | --- | --- | --- | --- | --- | --- | --- | --- |
|  |  |  | Q1 | Q2 | | Q3 | | Q4 | | Q5 | Q1 | Q2 | | Q3 | | Q4 | | Q5 |
| ANC visit in 1st Trimester |  |  | 0.455 | 0.752 | | 0.827 | | 0.884 | | 0.954 | 0.454 | 0.746 | | 0.813 | | 0.851 | | 0.929 |
| ANC visit in 2nd Trimester |  |  | 0.643 | 0.939 | | 0.971 | | 0.990 | | 0.996 | 0.620 | 0.931 | | 0.970 | | 0.979 | | 0.994 |
| ANC visits in 3rd Trimester | 1 |  | 0.035 | 0.005 | | 0.002 | | 0.001 | | 0.000 | 0.038 | 0.006 | | 0.001 | | 0.002 | | 0.000 |
|  | 2 |  | 0.532 | 0.880 | | 0.929 | | 0.964 | | 0.986 | 0.485 | 0.864 | | 0.935 | | 0.958 | | 0.988 |
|  | None |  | 0.433 | 0.116 | | 0.069 | | 0.036 | | 0.014 | 0.478 | 0.129 | | 0.064 | | 0.041 | | 0.012 |
| Weight measured during ANC |  |  | 0.584 | 0.949 | | 0.987 | | 0.996 | | 1.000 | 0.562 | 0.937 | | 0.977 | | 0.992 | | 0.999 |
| Height measured during ANC |  |  | 0.138 | 0.319 | | 0.435 | | 0.615 | | 0.830 | 0.136 | 0.326 | | 0.425 | | 0.545 | | 0.766 |
| Blood Pressure measured during ANC |  |  | 0.642 | 0.960 | | 0.988 | | 0.997 | | 0.999 | 0.616 | 0.959 | | 0.979 | | 0.992 | | 0.998 |
| Urine sample taken during ANC |  |  | 0.101 | 0.260 | | 0.415 | | 0.591 | | 0.856 | 0.101 | 0.273 | | 0.384 | | 0.526 | | 0.782 |
| Blood sample taken during ANC |  |  | 0.135 | 0.266 | | 0.354 | | 0.531 | | 0.821 | 0.140 | 0.271 | | 0.362 | | 0.469 | | 0.717 |
| Stomach examined during ANC |  |  | 0.747 | 0.983 | | 0.992 | | 0.997 | | 0.999 | 0.727 | 0.979 | | 0.992 | | 0.994 | | 0.998 |
| Consultation during ANC |  |  | 0.440 | 0.797 | | 0.882 | | 0.938 | | 0.977 | 0.423 | 0.777 | | 0.866 | | 0.923 | | 0.966 |
| Received MNCH book during ANC |  |  | 0.419 | 0.772 | | 0.870 | | 0.929 | | 0.964 | 0.399 | 0.762 | | 0.849 | | 0.905 | | 0.959 |
| Iron Supplementation during pregnancy |  | | 0.399 | 0.701 | | 0.801 | | 0.881 | | 0.954 | 0.379 | 0.686 | | 0.787 | | 0.856 | | 0.936 |
|  |  |  |  |  | |  | |  | |  |  |  | |  | |  | |  |
|  |  |  |  |  | |  | |  | |  |  |  | |  | |  | |  |
|  |  |  |  |  | |  | |  | |  |  |  | |  | |  | |  |
|  |  |  |  |  | |  | |  | |  |  |  | |  | |  | |  |
|  |  |  |  |  | |  | |  | |  |  |  | |  | |  | |  |
| Tetanus Immunisation | Full Protection | | 0.319 | 0.574 | | 0.682 | | 0.749 | | 0.841 | 0.260 | 0.528 | | 0.660 | | 0.744 | | 0.885 |
|  | Partial Protection | | 0.171 | 0.207 | | 0.179 | | 0.156 | | 0.119 | 0.181 | 0.232 | | 0.191 | | 0.157 | | 0.088 |
|  | None |  | 0.510 | 0.219 | | 0.139 | | 0.095 | | 0.040 | 0.558 | 0.240 | | 0.149 | | 0.099 | | 0.027 |
| Pregnancy complication Advice | Symptoms only | | 0.019 | 0.039 | | 0.022 | | 0.021 | | 0.015 | 0.013 | 0.039 | | 0.032 | | 0.021 | | 0.013 |
|  | Symptoms and Help | | 0.103 | 0.334 | | 0.509 | | 0.675 | | 0.849 | 0.054 | 0.213 | | 0.414 | | 0.702 | | 0.941 |
|  | None |  | 0.878 | 0.627 | | 0.469 | | 0.304 | | 0.136 | 0.932 | 0.748 | | 0.554 | | 0.277 | | 0.046 |
| Discussed place of delivery during pregnancy |  |  | 0.374 | 0.747 | | 0.933 | | 0.983 | | 0.998 | 0.356 | 0.719 | | 0.902 | | 0.968 | | 0.995 |
| Discussed transportation to place of delivery during pregnancy | | | 0.131 | 0.429 | | 0.681 | | 0.847 | | 0.954 | 0.124 | 0.398 | | 0.637 | | 0.800 | | 0.930 |
| Discussed who would assist delivery during pregnancy | |  | 0.401 | 0.733 | | 0.919 | | 0.976 | | 0.996 | 0.390 | 0.707 | | 0.880 | | 0.962 | | 0.994 |
| Discussed payment for delivery during pregnancy |  |  | 0.348 | 0.677 | | 0.868 | | 0.946 | | 0.980 | 0.334 | 0.659 | | 0.828 | | 0.921 | | 0.974 |
| Discussed possible blood donor during pregnancy | |  | 0.016 | 0.044 | | 0.118 | | 0.217 | | 0.515 | 0.017 | 0.054 | | 0.121 | | 0.202 | | 0.402 |
| Baby was weighed at birth |  |  | 0.581 | 0.897 | | 0.954 | | 0.978 | | 0.997 | 0.571 | 0.872 | | 0.947 | | 0.971 | | 0.990 |
| Baby was breastfed within 1 hr of birth |  |  | 0.388 | 0.403 | | 0.453 | | 0.552 | | 0.686 | 0.431 | 0.459 | | 0.462 | | 0.496 | | 0.565 |
| No liquids given before milk began to flow (no prelacteal feed) | | |  |  | |  | |  | |  |  |  | |  | |  | |  |
| Maternal postnatal check | Full (<2hrs) | | 0.248 | 0.411 | | 0.497 | | 0.574 | | 0.728 | 0.230 | 0.404 | | 0.476 | | 0.560 | | 0.696 |
|  | Partial (3+ hrs) | 3-12 hrs | 0.364 | 0.464 | | 0.448 | | 0.395 | | 0.261 | 0.353 | 0.454 | | 0.462 | | 0.404 | | 0.296 |
|  |  | 13-24hrs |  |  | |  | |  | |  |  |  | |  | |  | |  |
|  |  | 25-48hrs |  |  | |  | |  | |  |  |  | |  | |  | |  |
|  |  | 49hrs + |  |  | |  | |  | |  |  |  | |  | |  | |  |
|  | None |  | 0.389 | 0.126 | | 0.054 | | 0.032 | | 0.011 | 0.417 | 0.142 | | 0.062 | | 0.036 | | 0.009 |
| Neonatal postnatal check | Full (<2hrs) | | 0.102 | 0.204 | | 0.289 | | 0.396 | | 0.570 | 0.088 | 0.186 | | 0.275 | | 0.375 | | 0.538 |
|  | Partial (3+ hrs) | 3-12 hrs | 0.280 | 0.388 | | 0.389 | | 0.392 | | 0.313 | 0.256 | 0.370 | | 0.385 | | 0.396 | | 0.361 |
|  |  | 13-24hrs |  |  | |  | |  | |  |  |  | |  | |  | |  |
|  |  | 25-48hrs |  |  | |  | |  | |  |  |  | |  | |  | |  |
|  |  | 49hrs + |  |  | |  | |  | |  |  |  | |  | |  | |  |
|  | None |  | 0.617 | 0.409 | | 0.322 | | 0.212 | | 0.117 | 0.656 | 0.443 | | 0.340 | | 0.229 | | 0.101 |
| Postpartum Vitamin A within 2 months of delivery | |  | 0.219 | 0.355 | | 0.467 | | 0.604 | | 0.782 | 0.225 | 0.357 | | 0.458 | | 0.548 | | 0.724 |

**Table S2.11:** Mean Indicator Values by Quintile – Scenario 8 DHS Indicators, 3 categories Any Iron

| Indicator |  |  | EW Quintile | |  | |  | |  | | PCA Quintile | |  | |  | |  | |
| --- | --- | --- | --- | --- | --- | --- | --- | --- | --- | --- | --- | --- | --- | --- | --- | --- | --- | --- |
|  |  |  | Q1 | Q2 | | Q3 | | Q4 | | Q5 | Q1 | Q2 | | Q3 | | Q4 | | Q5 |
| ANC visit in 1st Trimester |  |  | 0.427 | 0.747 | | 0.836 | | 0.899 | | 0.961 | 0.467 | 0.752 | | 0.808 | | 0.850 | | 0.916 |
| ANC visit in 2nd Trimester |  |  |  |  | |  | |  | |  |  |  | |  | |  | |  |
| ANC visits in 3rd Trimester | 1 |  |  |  | |  | |  | |  |  |  | |  | |  | |  |
|  | 2 |  |  |  | |  | |  | |  |  |  | |  | |  | |  |
|  | None |  |  |  | |  | |  | |  |  |  | |  | |  | |  |
| Weight measured during ANC |  |  |  |  | |  | |  | |  |  |  | |  | |  | |  |
| Height measured during ANC |  |  |  |  | |  | |  | |  |  |  | |  | |  | |  |
| Blood Pressure measured during ANC |  |  | 0.651 | 0.959 | | 0.987 | | 0.995 | | 0.999 | 0.637 | 0.950 | | 0.972 | | 0.989 | | 0.996 |
| Urine sample taken during ANC |  |  | 0.067 | 0.240 | | 0.394 | | 0.617 | | 0.859 | 0.091 | 0.282 | | 0.400 | | 0.528 | | 0.766 |
| Blood sample taken during ANC |  |  | 0.100 | 0.243 | | 0.374 | | 0.548 | | 0.796 | 0.121 | 0.283 | | 0.401 | | 0.489 | | 0.666 |
| Stomach examined during ANC |  |  |  |  | |  | |  | |  |  |  | |  | |  | |  |
| Consultation during ANC |  |  |  |  | |  | |  | |  |  |  | |  | |  | |  |
| Received MNCH book during ANC |  |  |  |  | |  | |  | |  |  |  | |  | |  | |  |
| Iron Supplementation during pregnancy |  | | 0.347 | 0.711 | | 0.825 | | 0.887 | | 0.965 | 0.351 | 0.714 | | 0.781 | | 0.863 | | 0.936 |
|  |  |  |  |  | |  | |  | |  |  |  | |  | |  | |  |
|  |  |  |  |  | |  | |  | |  |  |  | |  | |  | |  |
|  |  |  |  |  | |  | |  | |  |  |  | |  | |  | |  |
|  |  |  |  |  | |  | |  | |  |  |  | |  | |  | |  |
|  |  |  |  |  | |  | |  | |  |  |  | |  | |  | |  |
| Tetanus Immunisation | Full Protection | | 0.278 | 0.585 | | 0.687 | | 0.725 | | 0.893 | 0.197 | 0.546 | | 0.659 | | 0.753 | | 0.922 |
|  | Partial Protection | | 0.198 | 0.188 | | 0.174 | | 0.194 | | 0.075 | 0.212 | 0.226 | | 0.199 | | 0.154 | | 0.058 |
|  | None |  | 0.524 | 0.227 | | 0.140 | | 0.081 | | 0.033 | 0.590 | 0.228 | | 0.142 | | 0.093 | | 0.020 |
| Pregnancy complication Advice | Symptoms only | | 0.021 | 0.029 | | 0.023 | | 0.031 | | 0.012 | 0.013 | 0.031 | | 0.037 | | 0.026 | | 0.011 |
|  | Symptoms and Help | | 0.105 | 0.324 | | 0.495 | | 0.665 | | 0.844 | 0.017 | 0.125 | | 0.414 | | 0.780 | | 0.989 |
|  | None |  | 0.874 | 0.647 | | 0.482 | | 0.304 | | 0.144 | 0.970 | 0.844 | | 0.548 | | 0.194 | | 0.000 |
| Discussed place of delivery during pregnancy |  |  |  |  | |  | |  | |  |  |  | |  | |  | |  |
| Discussed transportation to place of delivery during pregnancy | | |  |  | |  | |  | |  |  |  | |  | |  | |  |
| Discussed who would assist delivery during pregnancy | |  |  |  | |  | |  | |  |  |  | |  | |  | |  |
| Discussed payment for delivery during pregnancy |  |  |  |  | |  | |  | |  |  |  | |  | |  | |  |
| Discussed possible blood donor during pregnancy | |  |  |  | |  | |  | |  |  |  | |  | |  | |  |
| Baby was weighed at birth |  |  | 0.579 | 0.899 | | 0.955 | | 0.982 | | 0.994 | 0.584 | 0.879 | | 0.936 | | 0.963 | | 0.989 |
| Baby was breastfed within 1 hr of birth |  |  | 0.331 | 0.377 | | 0.468 | | 0.554 | | 0.746 | 0.419 | 0.435 | | 0.483 | | 0.503 | | 0.574 |
| No liquids given before milk began to flow (no prelacteal feed) | | |  |  | |  | |  | |  |  |  | |  | |  | |  |
| Maternal postnatal check | Full (<2hrs) | | 0.212 | 0.399 | | 0.487 | | 0.583 | | 0.764 | 0.179 | 0.377 | | 0.493 | | 0.552 | | 0.765 |
|  | Partial (3+ hrs) | 3-12 hrs | 0.386 | 0.488 | | 0.459 | | 0.383 | | 0.231 | 0.391 | 0.495 | | 0.440 | | 0.412 | | 0.230 |
|  |  | 13-24hrs |  |  | |  | |  | |  |  |  | |  | |  | |  |
|  |  | 25-48hrs |  |  | |  | |  | |  |  |  | |  | |  | |  |
|  |  | 49hrs + |  |  | |  | |  | |  |  |  | |  | |  | |  |
|  | None |  | 0.403 | 0.113 | | 0.054 | | 0.033 | | 0.005 | 0.430 | 0.127 | | 0.067 | | 0.036 | | 0.005 |
| Neonatal postnatal check | Full (<2hrs) | | 0.072 | 0.189 | | 0.278 | | 0.372 | | 0.634 | 0.047 | 0.169 | | 0.306 | | 0.351 | | 0.591 |
|  | Partial (3+ hrs) | 3-12 hrs | 0.273 | 0.398 | | 0.410 | | 0.411 | | 0.276 | 0.227 | 0.389 | | 0.391 | | 0.411 | | 0.349 |
|  |  | 13-24hrs |  |  | |  | |  | |  |  |  | |  | |  | |  |
|  |  | 25-48hrs |  |  | |  | |  | |  |  |  | |  | |  | |  |
|  |  | 49hrs + |  |  | |  | |  | |  |  |  | |  | |  | |  |
|  | None |  | 0.656 | 0.413 | | 0.311 | | 0.217 | | 0.089 | 0.726 | 0.442 | | 0.303 | | 0.237 | | 0.060 |
| Postpartum Vitamin A within 2 months of delivery | |  | 0.145 | 0.325 | | 0.492 | | 0.630 | | 0.821 | 0.156 | 0.377 | | 0.476 | | 0.563 | | 0.741 |
